# Temporal Variability of *Escherichia coli* Diversity in the Gastrointestinal Tracts of Tanzanian Children with and without Exposure to Antibiotics

**DOI:** 10.1128/mSphere.00558-18

**Published:** 2018-11-07

**Authors:** Taylor K. S. Richter, Tracy H. Hazen, Diana Lam, Christian L. Coles, Jessica C. Seidman, Yaqi You, Ellen K. Silbergeld, Claire M. Fraser, David A. Rasko

**Affiliations:** a The Institute for Genome Sciences, University of Maryland School of Medicine, Baltimore, Maryland, USA; b Department of Microbiology and Immunology, University of Maryland School of Medicine, Baltimore, Maryland, USA; c Department of Medicine, University of Maryland School of Medicine, Baltimore, Maryland, USA; d Department of International Health, Johns Hopkins Bloomberg School of Public Health, Baltimore, Maryland, USA; e Division of International Epidemiology and Population Studies, Fogarty International Center, National Institutes of Health, Bethesda, Maryland, USA; f Department of Environmental Health, Johns Hopkins Bloomberg School of Public Health, Baltimore, Maryland, USA; Antimicrobial Development Specialists, LLC

**Keywords:** *Escherichia coli*, diversity, microbial genomics

## Abstract

The stability of the Escherichia coli populations in the human gastrointestinal tract is not fully appreciated, and represents a significant knowledge gap regarding gastrointestinal community structure, as well as resistance to incoming pathogenic bacterial species and antibiotic treatment. The current study examines the genomic content of 240 Escherichia coli isolates from 30 children, aged 2 to 35 months old, in Tanzania. The E. coli strains were isolated from three time points spanning a six-month time period, with and without antibiotic treatment. The resulting isolates were sequenced, and the genomes compared. The findings in this study highlight the transient nature of E. coli strains in the gastrointestinal tract of these children, as during a six-month interval, no one individual contained phylogenomically related isolates at all three time points. While the majority of the isolates at any one time point were phylogenomically similar, most individuals did not contain phylogenomically similar isolates at more than two time points. Examination of global genome content, canonical E. coli virulence factors, multilocus sequence type, serotype, and antimicrobial resistance genes identified diversity even among phylogenomically similar strains. There was no apparent increase in the antimicrobial resistance gene content after antibiotic treatment. The examination of the E. coli from longitudinal samples from multiple children in Tanzania provides insight into the genomic diversity and population variability of resident E. coli within the rapidly changing environment of the gastrointestinal tract of these children.

**IMPORTANCE** This study increases the number of resident Escherichia coli genome sequences, and explores E. coli diversity through longitudinal sampling. We investigate the genomes of E. coli isolated from human gastrointestinal tracts as part of an antibiotic treatment program among rural Tanzanian children. Phylogenomics demonstrates that resident E. coli are diverse, even within a single host. Though the E. coli isolates of the gastrointestinal community tend to be phylogenomically similar at a given time, they differed across the interrogated time points, demonstrating the variability of the members of the E. coli community in these subjects. Exposure to antibiotic treatment did not have an apparent impact on the E. coli community or the presence of resistance and virulence genes within E. coli genomes. The findings of this study highlight the variable nature of specific bacterial members of the human gastrointestinal tract.

## Abstract (precis)

This study increases the number of resident Escherichia coli genome sequences, and explores E. coli diversity through longitudinal sampling. We investigate the genomes of E. coli isolated from human gastrointestinal tracts as part of an antibiotic treatment program among rural Tanzanian children. Phylogenomics demonstrates that resident E. coli are diverse, even within a single host. Though the E. coli isolates of the gastrointestinal community tend to be phylogenomically similar at a given time, they differed across the interrogated time points, demonstrating the variability of the members of the E. coli community in these subjects. Exposure to antibiotic treatment did not have an apparent impact on the E. coli community or the presence of resistance and virulence genes within E. coli genomes. The findings of this study highlight the variable nature of specific bacterial members of the human gastrointestinal tract.

## INTRODUCTION

Escherichia coli in the human gastrointestinal tract is often recognized as an important source of disease (1, 2). As the causative agent of over 2 million deaths annually due to diarrhea (3, 4), as well as millions of extraintestinal infections (5), its categorization as a pathogen is not unwarranted. Particularly in developing countries, the consequences of diarrheal E. coli are substantial among children under 5 years old, who incur the majority of infections and deaths (3) and whose rapidly developing microbiomes can be impacted by frequent bouts of disease and subsequent treatment (6, 7). Yet, E. coli is a dominant organism in the human gastrointestinal tract, identified in greater than 90% of humans, and many other large mammals, often reaching concentrations up to 10^9^ CFU per gram of feces (8) without causing disease. In this role as a resident organism in healthy hosts, it is thought to have critical roles in digestion, nutrition, metabolism, and protection against incoming enteric pathogens (9–12). Despite the importance and involvement of E. coli in human health, studies of its role as a native, nonpathogenic member of the human gastrointestinal microbiome are poorly represented among genome sequencing, comparative analysis efforts and functional characterization.

Investigations into E. coli strain diversity and persistence in the human gastrointestinal tract are nothing new. In fact, studies going back to 1899 (13) have reported on fecal E. coli diversity and persistence. Additional studies have continued to probe this question with the advent of new microbiological technologies beginning with antigenic techniques (13, 14), electrophoresis (15, 16), and PCR (17), to name a few. Today, thanks to the ready access of whole-genome sequencing, we have an unprecedented opportunity to explore E. coli diversity and persistence at the genomic level. Most studies of bacterial genomics have focused on pathogenic isolates over a limited time frame. E. coli genomic studies are no exception, having concentrated on sequencing single isolates, from single time points, and on samples related to a clinical presentation, such as diarrhea or urinary tract infection (10, 18–22). There have been fewer than five closed genomes sequenced of nonpathogenic E. coli, in addition to a limited number of draft genomes from isolates obtained from the feces of individuals who do not have diarrhea (10, 22–25). To date, the genomic examination of longitudinal isolates is lacking, thus hindering the ability to explore the diversity of E. coli isolates both within host and across time. With the exception of Stoesser et al. (23), which identified multiple isolates in single-host samples using single nucleotide polymorphism (SNP)-level analyses, most studies of resident E. coli were completed prior to ready access to sequencing technologies (11), leaving much to be learned about E. coli genomic diversity within and between human hosts over longitudinal sampling.

**FIG 1:**
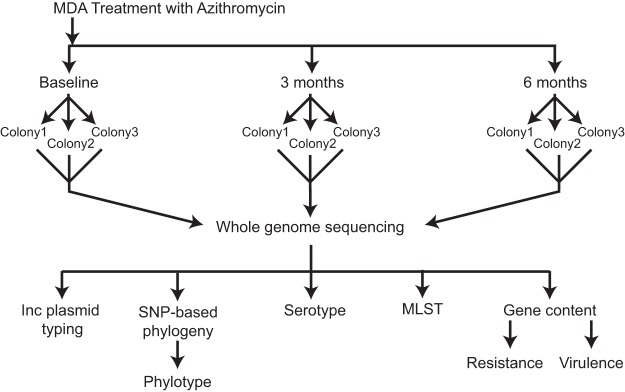
Overall study design. The overall design of the study highlighting the sampling of up to three distinct colonies on three time points, one of which, termed the baseline, occurs prior to the administration of antibiotics in half of the subjects.

A population-based longitudinal cohort study, PRET+ (Partnership for the Rapid Elimination of Trachoma, January to July 2009), provided a unique opportunity to examine both the diversity and dynamics of the E. coli isolates in the human gastrointestinal tract among children in rural Tanzania (26, 27). In the PRET+ study, Seidman et al. investigated the effects of mass distribution of azithromycin on antibiotic resistance of resident E. coli (26, 27). E. coli bacteria were isolated from fecal swabs obtained from 30 children aged 2 to 35 months old living in rural Tanzania, half (15 children) of whom were given a single oral prophylactic azithromycin treatment for trachoma (an infection of the eye caused by Chlamydia trachomatis). E. coli isolates from this cohort were selected for genome sequencing and comparative analyses to investigate the within-subject and longitudinal diversity of E. coli isolates in children (see Table S1 in the supplemental material). Up to three isolates per individual, from each of three time points spanning six months, were collected in the PRET+ study, providing up to nine potential isolates from each subject for examination (Fig. 1).

10.1128/mSphere.00558-18.4 TABLE S1 Subject and isolate information. Download Table S1, PDF file, 0.1 MB. Copyright © 2018 Richter et al. 2018 Richter et al. This content is distributed under the terms of the Creative Commons Attribution 4.0 International license.

Samples from the current study provide insight into E. coli diversity within a subject over several time points. While other studies have examined resident E. coli in children in developing countries, they limited their focus to using PCR and *in vitro* lab techniques to identify a limited set of canonical virulence genes and determine resistance profiles of the isolated strains (28–30). In addition to the virulence- and resistance-associated gene content, the current study demonstrates previously uncharacterized diversity among E. coli isolates from the human gastrointestinal tract on a whole-genome level within and across sampling periods. This work represents the most comprehensive longitudinal genomic study of resident E. coli within the human gastrointestinal tract and expands knowledge of the nonpathogen gut flora by increasing the available genome sequences of resident E. coli and highlighting the dynamic nature of the E. coli community.

## RESULTS

### Selection of E. coli strains for genome sequencing.

**FIG 2:**
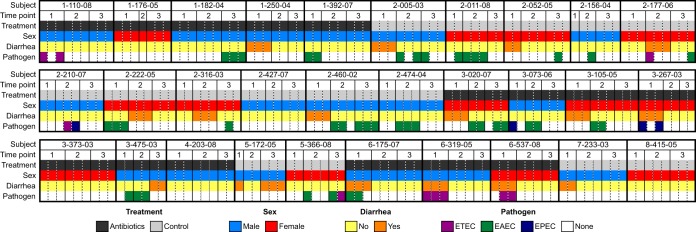
Isolate metadata. Summary of metadata showing time point of isolation, treatment group, host sex, clinical presentation, and the identification of pathogenic markers for ETEC, EAEC, or EPEC pathotypes for each isolate by subject. Further details in Table S1.

A total of 247 E. coli isolates from 30 subjects (17 male and 13 female as shown in Fig. 2) in the study by Seidman et al. (26, 27) were selected for DNA extraction and genome assembly, based on the criteria that these subjects contributed the most complete longitudinal collection of isolates (i.e., the greatest number of subjects with the greatest number of possible isolates). Of these, 240 isolates provided acceptable sequence quality to generate genome assemblies with a genome size and GC content that is characteristic of E. coli to be analyzed using comparative genomics. The average genome size was 5.17 Mb (range 4.46 to 5.81 Mb) with a 50.69% GC content (range 50.21 to 51.04%), similar to other known E. coli genomes (see Table S1 in the supplemental material). Of the 240 isolates, 120 isolates were from the subjects who received the antibiotic treatment of a single oral dose of prophylactic azithromycin, and 120 isolates were from subjects in the nontreatment (control) group (Table S1 and Fig. 2).

### Subject clinical state and E. coli pathotype identification.

There were 17 instances in which subjects had active diarrhea at the time of sample collection (12 instances occurred at the baseline time point), yielding 46 isolates from diarrheal conditions (26, 27), 23 each from the antibiotic treatment and control groups. All cases of diarrhea were identified in children under the age of 2. Only 10 of these isolates (21.7%) contained canonical virulence factors belonging to the EPEC (3 isolates), ETEC (6 isolates), or EAEC (1 isolate) pathotypes (Fig. 2), as determined by sequence homology searches of canonical virulence genes in the assembled genomes. In most cases, observed diarrhea could not be associated with a prototypically virulent E. coli strain in this data set. Other sources of diarrhea were not investigated.

An additional 61 isolates from 19 individuals contained canonical E. coli virulence factors, but were not obtained from samples taken during an active diarrheal event. These data indicate that the presence of a potentially virulent E. coli strain does not necessarily result in clinical presentation of diarrhea. Overall, in our data set association between diarrheal cases and incidence of isolates containing canonical E. coli virulence factors was rare.

### Phylogenomic analysis.

**FIG 3:**
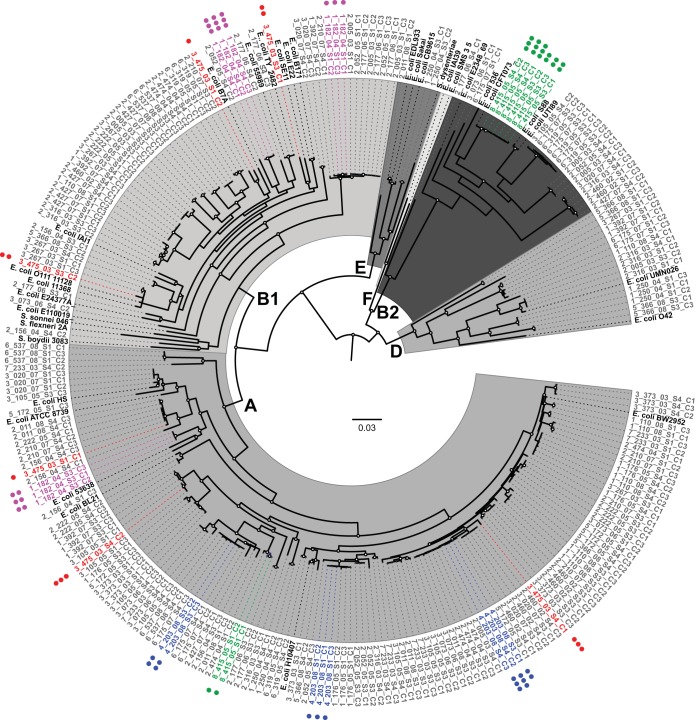
Phylogenomic analysis of E. coli isolates in study. A whole-genome phylogeny of the isolate sequences and reference E. coli and *Shigella* genomes (shown in black) highlighting examples of diversity among subject-specific isolates within and across time points. The scale bar indicates the approximate distance of 0.03 nucleotide substitutions per site. Nodes with bootstrap values of greater than 90 are marked with a circle. Examples of isolates from subjects that demonstrate the greatest (3_475_03) and least (4_203_08, 8_415_05, and 1_182_04) amount of diversity are highlighted: 3_475_03 in red, 4_203_08 in blue, 8_415_05 in green, and 1_182_04 in purple. The number of dots denotes the sample number from which the isolate was obtained. E. coli phylogroups are labeled. A full figure with all subjects is presented in Fig. S1.

Phylogenomic analysis of the isolates identified a diverse population of E. coli within the gastrointestinal community of these children. A phylogenetic tree of the 240 isolates from this study plus 33 reference E. coli and *Shigella* genomes (Table S2) was used to assess the genomic similarity of the isolates from a single subject both within and across time points, as well as between subjects over the study period (Fig. 3). The SNP-based phylogenomic analysis of the draft and reference genomes identified 304,497 polymorphic single nucleotide genomic sites. The isolates from the current study were identified in the established E. coli phylogroups: A (132 isolates), B1 (62 isolates), B2 (24 isolates), D (17 isolates), and E (2 isolates) (Fig. 3 and Table S1). Additionally, three isolate genomes (isolates 1_176_05_S3_C2, 2_011_08_S1_C1, and 2_156_04_S3_C2) fell into cryptic clades located outside the established E. coli phylogroups. The distributions of the E. coli isolates in each of these phylogroups were not associated with any of the clinical parameters associated with these isolates.

10.1128/mSphere.00558-18.5 TABLE S2 Reference genomes and corresponding pathotypes. Download Table S2, PDF file, 0.04 MB. Copyright © 2018 Richter et al. 2018 Richter et al. This content is distributed under the terms of the Creative Commons Attribution 4.0 International license.

10.1128/mSphere.00558-18.1 FIG S1 Distribution of the isolates in the phylogeny based on the sampling period. A cladogram of the phylogeny highlighting relative positions of all the isolate genomes from each subject with time point 1 in yellow, 2 in orange, and 3 in red. Download FIG S1, TIF file, 1.0 MB. Copyright © 2018 Richter et al. 2018 Richter et al. This content is distributed under the terms of the Creative Commons Attribution 4.0 International license.

To further investigate the E. coli diversity of an individual subject at a given time, we analyzed the phylogenetic groupings of isolates from each subject at each time point. Most isolates from an individual at a single time point group together within a single phylogenomic lineage, where a lineage is defined as a terminal grouping of isolates (54.4%; 49 of the 90 same-subject time points). One-third (35.5%; 32/90 of the same-subject time point isolates) fell into two distinct lineages, and in 10% (9/90 time points), all isolates belonged to a distinct lineage (Table 1). Overall, these data suggest that while there is considerable diversity among the isolates from many of the subjects, in over half of them, the population of E. coli at a given time point displays limited phylogenomic variation. The relatedness of co-occurring isolates was further confirmed by comparing the total gene content of the genomes from each subject. Those genomes found in the same phylogenetic clade had fewer divergent genes when the genomes were compared (average of 147.9 ± 120.1) than those found in different clades (average of 2,629.1 ± 339.4) (Table S3), further confirming the relatedness of the isolates within each clade.

**TABLE 1:** Summary of isolate diversity within subject and within time points^*a*^

Subject ID	Treatment	Isolate phylogenomics		Resistance			Virulence			Phylogroup		MLST		Serotype	
		No. of isolates from subject	No. of clades in subject^*b*^	No. of resistance clades^*b*^	Isolates in single resistance superclade	Similar distribution as phylogeny^*b*^	No. of virulence gene clades^*b*^	Similar distribution as phylogeny^*b*^	Similar distribution as resistance genes^*b*^	No. of phylogroups in subject^*b*^	Similar distribution as phylogeny^*b*^	No. of sequence types in subject^*b*^	Similar distribution as phylogeny^*b*^	No. of serotypes in subject^*b*^	Similar distribution as phylogeny^*b*^
1_110_08	MDA	9	5	5	No	No	5	No	Yes	3	No	3	No	5	Yes
1_176_05	MDA	8	4	4	No	Yes	4	Yes	Yes	2	No	3	No	4	Yes
1_182_04	MDA	9	3	5	No	No	3	Yes	No	2	No	3	Yes	3	Yes
1_250_04	MDA	7	3	2	Yes	No	3	Yes	No	3	Yes	3	Yes	3	Yes
1_392_07	MDA	8	4	5	No	No	4	Yes	No	3	No	4	Yes	4	Yes
3_020_07	MDA	8	4	4	No	Yes	4	Yes	Yes	3	No	4	Yes	4	Yes
3_073_06	MDA	7	5	5	No	Yes	4	No	No	3	No	5	No	5	No
3_105_05	MDA	9	7	6	No	Yes	7	Yes	No	2	No	7	Yes	7	Yes
3_267_03	MDA	7	6	6	No	Yes	6	Yes	Yes	3	No	6	Yes	6	Yes
3_373_03	MDA	9	4	4	No	Yes	3	No	No	1	No	3	No	4	Yes
3_475_03	MDA	6	6	5	No	No	6	Yes	No	2	No	6	Yes	6	Yes
4_203_08	MDA	8	3	5	No	No	3	No	No	1	No	2	No	3	Yes
6_175_07	MDA	9	4	5	No	Yes	5	Yes	Yes	3	No	4	Yes	4	Yes
6_319_05	MDA	8	3	5	No	No	3	Yes	No	3	Yes	4	No	3	Yes
6_537_08	MDA	8	3	5	No	No	3	Yes	No	2	No	3	Yes	3	Yes
2_005_03	No MDA	9	5	7	No	No	6	Yes	No	5	Yes	5	Yes	4	No
2_011_08	No MDA	8	6	5	No	No	6	Yes	No	3	Yes	6	No	7	No
2_052_05	No MDA	8	5	4	No	Yes	5	No	No	3	No	5	Yes	5	Yes
2_156_04	No MDA	7	7	5	No	Yes	6	No	No	2	No	6	No	7	Yes
2_177_06	No MDA	9	6	5	No	No	7	No	No	3	No	6	Yes	6	Yes
2_210_07	No MDA	8	6	6	No	Yes	5	No	No	2	No	6	Yes	6	Yes
2_222_05	No MDA	9	4	5	No	No	4	Yes	No	2	No	4	Yes	4	Yes
2_316_03	No MDA	8	6	7	No	No	5	No	No	3	No	6	Yes	5	No
2_427_07	No MDA	8	5	4	No	Yes	6	No	No	3	No	5	No	7	No
2_460_02	No MDA	9	4	4	No	Yes	4	Yes	Yes	3	No	4	Yes	5	No
2_474_04	No MDA	8	4	3	No	No	3	No	Yes	2	No	4	Yes	4	Yes
5_172_05	No MDA	6	4	3	No	No	4	Yes	No	1	No	4	Yes	4	Yes
5_366_08	No MDA	7	5	5	No	Yes	4	No	No	3	No	6	No	5	Yes
7_233_03	No MDA	8	5	5	Yes	Yes	5	Yes	Yes	1	No	4	No	6	No
8_415_05	No-MDA	8	2	3	No	No	2	Yes	No	2	Yes	3	No	2	Yes

a Diversity is measured using phylogenomics, resistance gene profiles, virulence gene profiles, phylogroups, MLST, and serotype. Cladograms were used to determine the relationships in the resistance gene profiles and virulence gene profiles of isolates within a subject and the number of lineages within each subject. Lineages with similar distributions are those that comprise the same isolates across diversity measurements. Phylogroups, MLST, and serotype distributions are considered similar if they contain the same number of types as phylogenomic lineages.

b Further details are provided in Table S3.

10.1128/mSphere.00558-18.6 TABLE S3 Table of pairwise gene content comparisons for each individual showing the relatedness of genomes within and across clades. Download Table S3, PDF file, 0.04 MB. Copyright © 2018 Richter et al. 2018 Richter et al. This content is distributed under the terms of the Creative Commons Attribution 4.0 International license.

These E. coli populations were variable over time, demonstrating increased E. coli diversity in each subject when observed over the multiple time points. Same-subject isolates from different time points resided in distinct phylogenomic lineages in 93.3% (28/30) of subjects, whereas more than half of the isolates from any individual at a single time point grouped together in a single lineage. Only two subjects had isolates from multiple time points that occupied the same lineage (subjects 4_203_08 and 8_415_05) (illustrated in Fig. 3 and detailed in Table S4). In contrast, all isolates from subject 3_475_03 were phylogenomically distinct (Fig. 3). These examples of the phylogenomic distributions of isolates represent the extremes of conservation or diversity that are observed with this study. Additional sampling will most likely reveal that the isolates within these individuals are not conserved or diverse as this initial sampling would suggest, but they do represent the possible distributions of the isolates within a subject over time.

10.1128/mSphere.00558-18.7 TABLE S4 Details of isolate diversity within subject and within time point across several diversity measurements. Download Table S4, PDF file, 0.1 MB. Copyright © 2018 Richter et al. 2018 Richter et al. This content is distributed under the terms of the Creative Commons Attribution 4.0 International license.

### Multilocus sequence typing and molecular serotyping.

**FIG 4:**
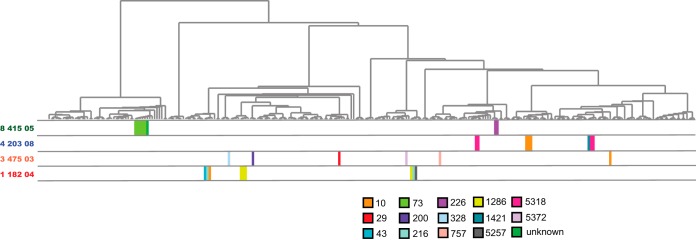
Phylogenomic distribution of sequence types of isolates from select subjects. A cladogram of the phylogeny highlighting relative positions of genomes of isolates from selected subjects with MLST sequence types shown in colored blocks corresponding to the sequence type as shown in the legend. Selected example subjects highlight low diversity within time points but high diversity across time (subject 1_182_04), high diversity within and across time (3_475_03), intermediate diversity across time (4_203_08), and low diversity across time (8_415_05).

The genomes in this study comprise a combined total of 87 sequence types (STs) (Table S1). The most common ST was ST10, which was represented by 40 of the E. coli genomes, while 40 additional STs occurred only once (Table S1). Only five isolates were from ST131, which has been demonstrated to be associated with the spread of antimicrobial resistance (31). There were, on average, 1.5 (range 1 to 3) STs among isolates from a subject at a single time point, and an average of 4.4 (range 2 to 7) STs per subject across all time points. Since the total number of available isolates per subject varied, the values were normalized per the number of isolates, revealing an average of 2 (range 1 to 4) isolates per sequence type and mimicking the diversity observed in the phylogenomic analyses (Fig. 4 and Table S4).

Similar to MLST, serotype analyses (32) reflect the diversity observed in the phylogenomic analysis (Table S4). The 240 isolates represent a combined total of 106 O:H serotypes, with 54 of them occurring only once in the data set, making serotype a finer-scale measure of diversity than MLST. There is an average of 1.63 (range 1 to 3) different serotypes in isolates from the same time point and 4.7 (range 2 to 7) serotypes in a subject across all time points. The O, H, or either serotype could not be predicted in 33 isolates (Table S1). *In silico* analyses were unable to distinguish between some serotypes in an additional 58 isolates (Table S1). This left 149 isolates that could be unambiguously assigned a single serotype (Table S1).

Nearly all isolates that shared a serotype also shared an MLST sequence type and phylogroup (Table S1). There are five examples (excluding those isolates in which the serotype could not be unambiguously differentiated) where MLST, serotype, and phylogroup were not congruent (Table S5), suggesting molecular variation and strain differentiation could not be detected by a single method alone. The combination of these detailed molecular methods could add nuance to diversity measurements in closely related strains.

10.1128/mSphere.00558-18.8 TABLE S5 Examples of isolates with identical serotypes, but differing sequence type and/or phylotype. Download Table S5, PDF file, 0.05 MB. Copyright © 2018 Richter et al. 2018 Richter et al. This content is distributed under the terms of the Creative Commons Attribution 4.0 International license.

### Genome content determined using LS-BSR.

Variations in genome content further demonstrated the diversity of the E. coli isolate genomes both within and between time points. Using the LS-BSR analysis (33) and an ergatis-based annotation pipeline, a gene content profile was determined which identified 32,950 genes in the pangenome of the 240 isolate genomes. More than 3,000 genes in any single genome were comprised of genes that vary between genomes, leaving only approximately 2,000 genes in the conserved core, as has been previously identified (10, 22). This level of variation is true even among the isolates from subject 8_415_05 in which the isolates from the 3-month and 6-month time points group together phylogenomically, and are of the same MLST sequence type. In this case, each isolate contains an average of 220 (range 95 to 259) variable genes. Given the level of diversity suggested by the variability of the gene content, more detailed SNP analyses, as previously performed by Stoesser et al. (23), were deemed unnecessary.

### Antibiotic resistance-associated gene profiles.

The antibiotic treatment of half of the children in this study provided a unique opportunity to investigate the impact of antibiotic treatment on the prevalence and maintenance of antibiotic resistance genes in the E. coli community at 3 and 6 months after administration. Antibiotic resistance genes were investigated in the isolate genomes using 1,371 genes from the Comprehensive Antibiotic Resistance Database (CARD) (34). The resistance gene profiles (assortment of present/absent genes) for each isolate were used to create a cladogram to investigate the relationships among isolates by time and by subject (Fig. S2). These relationships were then compared to those in the phylogenomic groupings as well as in the cladogram of virulence gene profiles (Table S6 and Fig. S3). Similar clustering patterns were identified between the whole-genome phylogeny or virulence gene presence and resistance gene-based analysis 74% of the time at each time point, and 37% (phylogeny) or 27% (virulence) of the time for each subject as a whole (Table 1).

10.1128/mSphere.00558-18.2 FIG S2 Interrogation of the antimicrobial resistance genes in the E. coli isolates. *In silico* detection of the resistance-associated genes from the CARD databases in the E. coli isolates. The colors of the heat map indicate the presence of each protein-encoding gene with significant similarity (yellow) or their absence (blue). Each column represents a different gene from the database, and each row/tree leaf is an isolate. Download FIG S2, EPS file, 1.6 MB. Copyright © 2018 Richter et al. 2018 Richter et al. This content is distributed under the terms of the Creative Commons Attribution 4.0 International license.

10.1128/mSphere.00558-18.3 FIG S3 Heat map and cladogram of distribution of virulence genes across isolates. Genes were identified using LS-BSR with high gene homology shown in yellow and low gene homology in blue. Each column represents a different gene from the database, and each row/tree leaf is an isolate. Cladogram is the hierarchical clustering of samples based on their virulence gene presence/absence. Download FIG S3, TIF file, 3.9 MB. Copyright © 2018 Richter et al. 2018 Richter et al. This content is distributed under the terms of the Creative Commons Attribution 4.0 International license.

10.1128/mSphere.00558-18.9 TABLE S6 LS-BSR of virulence genes in the isolate genomes. Download Table S6, XLSX file, 0.2 MB. Copyright © 2018 Richter et al. 2018 Richter et al. This content is distributed under the terms of the Creative Commons Attribution 4.0 International license.

There was no significant change in number or type of resistance-associated genes over time, regardless of antibiotic treatment or isolation time point. As subjects were treated with azithromycin, a macrolide, genes conferring resistance to macrolides were investigated in greater detail (Table S7). Macrolide resistance genes were identified in only 19% (46 of the 240) isolates (Table 2), and based on a logistic regression model, there is no evidence to suggest that either time point or antibiotic treatment was significantly associated with macrolide resistance genes (*P* > 0.05 for antibiotic treatment adjusted for time point, for time point adjusted for antibiotic treatment, and overall antibiotic treatment). Isolates from nearly half of the subjects had no known macrolide resistance genes (46.67% antibiotic treatment, 40% control). Based on these results, exposure to a single large dose of azithromycin did not lead to a significant change in the number of known antimicrobial resistance genes or macrolide resistance genes among these E. coli populations.

**TABLE 2:** Summary of macrolide resistance gene presence by treatment group and time point^*a*^

Time point(s) in which macrolide resistance genes found	Treatment					No treatment				
	Subject	% of isolates by time point (mo)			% (no. positive/no. total)	Subject	% of isolates by time point (mo)			% (no. positive/no. total)
		1	2	3			1	2	3	
No macrolide resistance genes	3_073_06	0	0	0	46.67 (7/15)	2_052_05	0	0	0	40 (6/15)
	3_373_03	0	0	0		2_156_04	0	0	0	
	3_475_03	0	0	0		2_177_06	0	0	0	
	4_203_08	0	0	0		2_222_05	0	0	0	
	6_175_07	0	0	0		2_474_04	0	0	0	
	6_319_05	0	0	0		8_415_05	0	0	0	
	6_537_08	0	0	0						
Only in 3 mo	1_176_05	0	0.5	1	13.33 (2/15)	2_005_03	0	0.66	0	33.33 (5/15)
	1_182_04	0	0.66	0		2_011_08	0	0.66	0	
						2_210_07	0	0.33	0	
						5_366_08	0	0.66	0	
						7_233_03	0	0.66	0	
Only in 6 mo	1_110_08	0	0	1	13.33 (2/15)	2_316_03	0	0	0.66	13.33 (2/15)
	1_392_07	0	0	0.66		2_427_07	0	0	0.66	
Pre- and posttreatment	1_250_04	1	1	1	13.33 (2/15)	2_460_02	0.66	0	1	6.67 (1/15)
	3_105_05	0.33	0.33	0.33						
3 and 6 mo	3_020_07	0	1	0.66	13.33 (2/15)					0.00
	3_267_03	0	0.5	0.5						
Only baseline					0.00	5_172_05	1	0	0	6.67 (1/15)

a The proportion of isolates in which a macrolide resistance gene was identified is shown for each time point. Subjects are separated in to treatment groups and categorized based on the time points in which macrolide resistance genes were identified. Percentages reflect the proportion of subjects who fall into each macrolide resistance gene category within treatment groups.

10.1128/mSphere.00558-18.10 TABLE S7 Presence of macrolide resistance genes. Download Table S7, PDF file, 0.1 MB. Copyright © 2018 Richter et al. 2018 Richter et al. This content is distributed under the terms of the Creative Commons Attribution 4.0 International license.

## DISCUSSION

This study represents a detailed examination of the genomic diversity of Escherichia coli isolates obtained from longitudinal samples from the gastrointestinal tract of children in rural Tanzania. An overall trend identified in this study is that the identified E. coli isolates from the gastrointestinal tract are diverse not just between these subjects, but within the same subject over time. The E. coli genomes sequenced in this study were selected based on the greatest number of longitudinal isolates per subject and include members of all five of the traditional E. coli phylogroups, as well as 87 different MLST sequence types, and 106 serotypes. The isolates in this study were most frequently of the A or B1 phylogroups, unlike a previous study by Gordon et al. (17) in which greater than 70% of the isolates obtained were from either phylogroup B2 or D. Other studies, featuring isolates from Europe and South America, have similarly identified phylogroup A as a dominant phylogroup in the human gastrointestinal tract (35, 36). This observed difference may be due to differences in sample acquisition (stool swab versus biopsy), differences in the study participants, or geography. The Gordon et al. (17) study obtained samples from adults, the majority (72.5%, 50/69) of whom were diagnosed with either Crohn’s disease or ulcerative colitis, which would also likely impact the immune status of the gastrointestinal tract, and potentially alter the bacterial community structure. In contrast, our study participants were children under the age of 5, and, other than a few who displayed diarrhea of an unknown source, were considered to be relatively healthy. This study, by using a combination of molecular methods, including whole-genome sequencing, enhances the understanding that E. coli in the human gastrointestinal tract is variable and diverse in the studied population.

Previous studies of the variability of E. coli, using non-genome sequencing methods, have also identified multiple isolates within a single host, reporting up to an average of 4 E. coli genotypes in adult human gastrointestinal studies (17, 23). The findings in this study are similar in that it has identified a number of E. coli isolates that are genomically and molecularly different in the subjects at each time and between time points. This study examines the relatedness of E. coli isolates in an individual over time using two independent methods, phylogenomics of the genome core and whole-genome content. We find that approximately half of E. coli isolates in an individual appear phylogenomically and phenotypically similar at any given time point; however, between time points, the prevalent E. coli clones from individual subjects were variable. While it is possible, and likely, that in the current study less prevalent E. coli isolates were not captured at some of the sampling time points, we assume that the relative isolate abundance in culture reflects the relative abundance in the feces at the time of sampling. The current study likely still underestimates the E. coli diversity in the examined subjects with the relatively small number of isolates collected per time point.

Dynamic populations within the human gastrointestinal tract have been previously suggested as an explanation for observations of variable clones in E. coli diversity studies (35), but the necessary longitudinal genomic studies were lacking. This study begins to address that deficiency, with the potential caveats outlined below. The observed within-patient and longitudinal diversity of E. coli isolates could be a function of age, as all of the subjects in this study were less than 3 years of age, and thus, the diversity could be a result of natural introduction of new exposure to foods, as well as immune system and microbiome development (37, 38). It has been demonstrated that intrahost E. coli diversity is greatest in tropical regions where hygiene may play a role and that E. coli density in the gastrointestinal tract is altered most significantly in the first 2 years of a child’s life (11, 39). Therefore, it is unclear how well these results correlate with E. coli diversity in adults or in other geographic regions, but they provide a starting point for the comparisons of studies in diverse subject populations and geographic locations. It is thought that the infant microbiome is not established until about 3 years of age (40); however, the detailed longitudinal infant microbiome studies are currently lacking. Furthermore, changes in health status may have impacted the strain variability, as some subjects displayed symptoms of diarrhea during sampling, with the possibility of other unreported occurrences between samples, leading to additional fluctuations in the E. coli community, as well as the potential emergence of otherwise rare, resident strains. Future longitudinal studies that include sampling subjects from multiple age groups will be necessary to fully appreciate levels of bacterial population diversity and dynamics present across host populations of all age groups.

Virulence and resistance-associated gene analyses in this study confirm that genomic analyses of single isolates are imperfect predictors of clinical phenotypes, as several isolates harbored canonical E. coli virulence genes, classically identifying them as enteric pathogens, but were present in subjects not displaying clinical symptoms. The converse is also possible, in that E. coli strains may not contain traditional virulence factors, but be obtained from a diarrheal sample, as has been highlighted in the recent GEMS studies (41, 42). While diarrheagenic E. coli is often the dominant strain when causing diarrhea (43), the fact that these pathogenic strains may have been missed due to undersampling in the diarrhea samples cannot be discounted. There are many potential explanations for these observations which include the following: (i) the subjects have been previously exposed to these bacteria, and thus, have an established immunity; (ii) the organisms are not pathogenic in the context of other host factors, including the host microbiota; (iii) additional necessary virulence factors are absent in these isolates; or (iv) the virulence factors are present but not expressed by the bacterium. Unfortunately, detailed immunological, microbiota, or transcriptional data are not available on the current samples, so the impacts of these factors on pathogenicity cannot be determined conclusively. Whole-genome analyses have led to increasing recognition that virulence genes and phylogeny are associated attributes in microbial pathogen genomes and suggest that there may be an optimal combination of chromosomal and virulence-associated features that results in maximal virulence, survival or transmission (44–47). This may also be true of the success of a commensal isolate in the community in these subjects (48).

In contrast to Seidman et al. (26), from which the samples were originally obtained, our genome analyses did not demonstrate an increase in the presence of macrolide resistance genes among isolates from children treated with azithromycin. This observation may be due to the selection of isolates for this genomic study. Subject samples sets with the greatest number of longitudinal isolates were chosen for sequencing. Additionally, genome sequencing did not include any samples from the first month after azithromycin treatment, which Seidman et al. found to demonstrate the greatest increase in phenotypic macrolide resistance (26). The examination of the 23S rRNA gene for SNPs associated with macrolide resistance is not possible due to the incomplete nature of the genomes and the genetic redundancy of the multiple copies of this gene cluster (49). This study, once again, highlights the discrepancies between genotypic and phenotypic assessment of resistance and other traits.

This study adds significantly to the number of available E. coli genomes that were not selected for based on pathogenic traits, a group that has been traditionally underrepresented in the sequencing of this species. The scientific community is still in the early stages of understanding gastrointestinal tract microbial ecology and the role that the resident bacteria, including E. coli, play in microbiome stability and function. The current study demonstrates that at the genomic level, the community of E. coli in the gastrointestinal tract of this population of children is diverse and variable over time. Further studies on human populations from different geographic areas, as well as other age groups, are required to determine if E. coli communities would stabilize as a person approaches adulthood, or whether the community diversity of E. coli regularly changes depending on the development of the immune system, as well as many other exposures within the gastrointestinal tract.

## MATERIALS AND METHODS

### Isolate selection.

E. coli isolates in this study were selected from isolates collected in Seidman et al. (26). The PRET+ study was a 6-month study designed to assess the ancillary effects on pneumonia, diarrhea and malaria in children following mass distribution of azithromycin for trachoma control. The study was conducted in 8 communities in the Kongwa, a district located in rural central Tanzania on a semiarid highland plateau with poor access to drinking water. The district has a total population of approximately 248,656, comprising mostly herders and subsistence farmers. The Tanzanian government stipulates that villages with trachoma prevalence ≥10% receive annual mass distribution of azithromycin. On survey, 4 villages found eligible for antibiotic treatment became the PRET+ treatment villages and 4 neighboring ineligible communities were included as controls. The study methods and results detailing the impact of antibiotic treatment on pneumonia and diarrhea morbidity and antibiotic-resistant Streptococcus pneumoniae carriage were published previously (50–52).

The selected E. coli isolates were chosen to represent individuals with the most complete longitudinal sample sets from the PRET+ E. coli substudy. Isolates were obtained from 30 individuals between 2 and 35 months of age, living in 8 villages in the same rural area of Tanzania. Half of these individuals received antibiotic treatment, while the other half (control) received no antibiotic treatment. These isolates were cultured from fecal samples collected at three time points (Fig. 1 and Table S1): a baseline prior to antibiotic treatment, three months posttreatment, and six months posttreatment, with corresponding time points in the untreated controls. A single treatment of 20 mg/kg of body weight of azithromycin was given 2 days after the baseline sample was collected. At each time point, up to three E. coli colonies per individual were selected for sequencing and subsequent comparative analyses. Isolates were labeled with a three-number subject ID (i.e., 1_110_08), the sample (time point) from which the isolate was obtained (i.e., S1), and the number of the colony isolated from the sample (i.e., C1).

### Bacterial growth and isolation.

E. coli colonies were obtained as described in Seidman et al. (26, 27). Briefly, fecal swabs were streaked on MacConkey agar (Difco) and grown overnight at 37°C. Three lactose fermentation (LF)-positive colonies were inoculated on nutrient agar stabs and grown overnight at 37°C. E. coli isolates were identified as those colonies which were LF-positive, indole-positive (DMACA Indole Reagent droppers, BD), and citrate-negative (Simmons citrate agar slants). Isolates were transferred to Luria broth for overnight growth at 37°C with shaking. E. coli cultures were frozen with 10% glycerol and stored at −80°C.

### Genome sequencing and assembly.

Genomic DNA was extracted using standard methods (21) and sequenced on the Illumina HiSeq 2000 platform at the Genome Resource Center at the University of Maryland School of Medicine, Institute for Genome Sciences (http://www.igs.umaryland.edu/resources/grc/). The resulting 100-bp reads were assembled as previously described (44, 46) using the Maryland Super-Read Celera Assembler (MaSuRCa version 2.3.2) (53). Contigs of fewer than 200 bp were excluded from assemblies. Assembly quality was determined based on number of contigs (less than 500), and genome size and G+C content compared to known E. coli genomes. Two genomes had G+C content divergent from that of E. coli (55.61%) and were excluded from further analysis. The assembly details and corresponding GenBank accession numbers are provided in Table S1.

### Identification of predicted pathogen isolates.

Isolate genomes were interrogated for the presence of pathotype-specific virulence factor genes using LS-BSR and are derived from a similar E. coli typing schema used in the MAL-ED studies (54). The nucleotide sequence for each factor or resistance gene was aligned against all sequenced genomes with BLASTN (55) in conjunction with LS-BSR (33). Genes with a BSR value ≥0.80 were considered highly conserved and present in the isolate examined. The targeted virulence factors are as follows: ETEC heat-stable enterotoxin (estA147) or ETEC heat-labile enterotoxin (eltb508), identifying the isolate as being enterotoxigenic E. coli (ETEC); the *aggR*-activated island C (aic215) or EAEC ABC transporter A (aata650) genes, which are common diagnostic markers for enteroaggregative E. coli (EAEC) (56, 57); and the major subunit of the bundle-forming pilus (*bfpA*) (bfpa300) or intimin genes (eae881), which are indicative of enteropathogenic E. coli (EPEC) (44).

### Phylogenomic analysis.

A total of 273 genomes were used in the phylogenomic analyses: the 240 assembled in this study, in addition to a collection of 33 E. coli and *Shigella* reference genomes from GenBank (Table S2). Single nucleotide polymorphisms (SNPs) in all genomes were detected relative to the completed genome sequence of commensal isolate E. coli HS (phylogroup A) using the *in silico* genotyper (ISG) v.0.12.2 (58), which uses MUMmer v.3.22 (59) for SNP detection. Analysis with ISG yielded 701,011 total SNP sites that were filtered to a subset of 304,497 SNP sites present in all of the genomes analyzed. These SNP sites were concatenated and used for phylogenetic analysis as previously described (60). A maximum-likelihood phylogeny with 1,000 bootstrap replicates was generated using RAxML v.7.2.8 (61) and visualized using FigTree v.1.4.2 (http://tree.bio.ed.ac.uk/software/figtree/) and interactive tree of life (62). Phylogenomic lineages were assigned based on visual determination of groupings. Three genome outliers (1_176_05_S3_C2, 2_011_08_S1_C1, and 2_156_04_S3_C2 were removed from the tree figures for visualization purposes.

### Serotype identification.

*In silico* serotype identification was performed on the assembled genomes using the online SerotypeFinder 1.1 (https://cge.cbs.dtu.dk/services/SerotypeFinder/) and an LS-BSR analysis using the serotype sequences compiled for the SRS2 program (https://github.com/katholt/srst2/tree/master/data) (20, 32).

### Multilocus sequence typing (MLST).

*In silico* MLST was performed on the assembled genomes using the Achtman E. coli MLST scheme (63). Gene sequences were identified in the isolate genomes using BLASTn, and MLST profiles were determined by querying the PubMLST database (http://pubmlst.org).

### Variations in gene distributions.

The gene content across all genomes was identified and compared using the large-scale BLAST score ratio (LS-BSR) with default settings, as previously described (33). Genes with a BSR value ≥0.80 are considered to be highly conserved and present in the isolate examined at this level of homology. Those genes that are conserved in all genomes were removed from further analyses. The predicted protein function of each gene cluster was determined using an Ergatis-based (64) in-house annotation pipeline (65).

Pairwise gene content comparisons were performed for all of the isolates for each subject to determine the number of genes that differed between the isolates. The numbers of differing genes were used to calculate the average number (and standard deviation) of genes that differed between isolates from the same phylogenomic clade and those from differing phylogenomic clades for each subject.

### Virulence factor and antibiotic resistance gene identification.

The list of compiled common E. coli virulence factors genes was used for interrogation of the study genomes (Table S2). Antibiotic resistance genes were compiled from the Comprehensive Antibiotic Resistance Database (CARD; http://arpcard.mcmaster.ca, downloaded 24 June 2015) (34). The nucleotide sequence for each factor or resistance gene was aligned against all sequenced genomes with BLASTN (55) in conjunction with LS-BSR (33). Genes with a BSR value ≥0.80 were considered highly conserved and present in the isolate examined.

### Statistical analysis of macrolide resistance gene distributions.

A logistic regression on the probability of a macrolide gene being present in an E. coli isolate was run against 2 covariates: time point (excluding the baseline) or antibiotic treatment. For each individual, the two to three isolates were considered replicates for that time point, and the time points were far enough apart to be considered independent. Therefore, gene presence was collapsed as presence in at least one of the replicates at a given subject and time point. Each subject by time combination was considered an independent observation. Genes in this analysis with *P* values ≤0.05 were considered significant. If the covariate was dichotomous, then the Wald chi-square test statistic was used to determine significance.
