# Supplementary material for: Temporal Variability of Escherichia coli Diversity in the Gastrointestinal Tracts of Tanzanian Children with and without Exposure to Antibiotics
Source: mSphere. 2018 Nov 7;3(6):e00558-18. doi: 10.1128/mSphere.00558-18 (PMC6222053; doi:10.1128/mSphere.00558-18)
Supplement: TABLE S1 [file sph006182690st1.pdf]

Table S1. Subject and isolate information

Subject and Isolate Data									Genome assembly information						Strain information		
Subject ID	MDA	Age	Sex	Diarrhea	Date	Time point	Time point ID	Colony	Assembly ID	WGS Accession	SRA Accession	Genome size	%GC	No. of contigs	MLST	Phylogroup	Serotype
1-110-08	Yes	2 yrs	M	no	1/13/09	baseline	S1	C1	1_110_08_S1_C1	JHDQ00000000	SRS567978	4910285	50.67	128	10	A	O159:H4
1-110-08	Yes	2 yrs	M	no	1/13/09	baseline	S1	C2	1_110_08_S1_C2	JHDP00000000	SRS568148	5010726	50.7	137	155	B1	O136:H25
1-110-08	Yes	2 yrs	M	no	1/13/09	baseline	S1	C3	1_110_08_S1_C3	JHDO00000000	SRS568343	4953256	50.71	133	10	A	O159:H4
1-110-08	Yes	2 yrs	M	no	4/11/09	3-month	S3	C1	1_110_08_S3_C1	JHDN00000000	SRS568156	5520284	50.42	189	10	A	O176:H33
1-110-08	Yes	2 yrs	M	no	4/11/09	3-month	S3	C2	1_110_08_S3_C2	JHDM00000000	SRS568140	5489145	50.41	176	10	A	O176:H33
1-110-08	Yes	2 yrs	M	no	4/11/09	3-month	S3	C3	1_110_08_S3_C3	JHDL00000000	SRS568168	5514291	50.42	175	10	A	O176:H33
1-110-08	Yes	2 yrs	M	no	7/4/09	6-month	S4	C1	1_110_08_S4_C1	JHDK00000000	SRS568248	5498100	50.53	159	38	D	O86:H18
1-110-08	Yes	2 yrs	M	no	7/4/09	6-month	S4	C2	1_110_08_S4_C2	JHDJ00000000	SRS568229	5219753	50.81	199	10	A	NT:H16
1-110-08	Yes	2 yrs	M	no	7/4/09	6-month	S4	C3	1_110_08_S4_C3	JHDI00000000	SRS568328	5200020	50.8	196	10	A	NT:H16
1-176-05	Yes	2 yrs	F	no	1/13/09	baseline	S1	C1	1_176_05_S1_C1	JHDH00000000	SRS568397	5132963	50.83	173	10	A	O107/O117:H10 Gp8
1-176-05	Yes	2 yrs	F	no	1/13/09	baseline	S1	C2	1_176_05_S1_C2	JJMA00000000	SRS568076	5151035	50.85	174	10	A	O107/O117:H10 Gp8
1-176-05	Yes	2 yrs	F	no	1/13/09	baseline	S1	C3	1_176_05_S1_C3	JJLY00000000	SRS568351	5178244	50.88	157	10	A	O107/O117:H10 Gp8
1-176-05	Yes	2 yrs	F	no	4/11/09	3-month	S3	C1	1_176_05_S3_C1	JHDG00000000	SRS568264	5327796	50.79	189	206	A	O101/O162:H5 Gp15
1-176-05	Yes	2 yrs	F	no	4/11/09	3-month	S3	C2	1_176_05_S3_C2	JHDF00000000	SRS568379	4457870	50.67	68	5792	cryptic	O13/O129/O135:H5 Gp10
1-176-05	Yes	2 yrs	F	no	7/3/09	6-month	S4	C1	1_176_05_S4_C1	JJLQ00000000	SRS568352	5179950	50.83	180	10	A	NT:H16
1-176-05	Yes	2 yrs	F	no	7/3/09	6-month	S4	C2	1_176_05_S4_C2	JMGW00000000	SRS568164	5065396	50.92	164	10	A	NT:H16
1-176-05	Yes	2 yrs	F	no	7/3/09	6-month	S4	C3	1_176_05_S4_C3	JJLI00000000	SRS568019	5168201	50.8	194	10	A	NT:H16
1-182-04	Yes	< 1 yr	M	no	1/13/09	baseline	S1	C1	1_182_04_S1_C1	JJMD00000000	SRS568367	5117656	50.64	89	23	B1	O8:H9
1-182-04	Yes	< 1 yr	M	no	1/13/09	baseline	S1	C2	1_182_04_S1_C2	JMGS00000000	SRS568340	4982681	50.54	114	23	B1	O8:H9
1-182-04	Yes	< 1 yr	M	no	1/13/09	baseline	S1	C3	1_182_04_S1_C3	JJLX00000000	SRS568142	5044280	50.56	89	23	B1	O8:H9
1-182-04	Yes	< 1 yr	M	no	4/11/09	3-month	S3	C1	1_182_04_S3_C1	JJLV00000000	SRS568331	5135834	50.83	306	5275	A	NT:H30
1-182-04	Yes	< 1 yr	M	no	4/11/09	3-month	S3	C2	1_182_04_S3_C2	JJLT00000000	SRS568249	4581474	50.89	261	5275	A	NT:H30
1-182-04	Yes	< 1 yr	M	no	4/11/09	3-month	S3	C3	1_182_04_S3_C3	JJLS00000000	SRS568158	5179152	50.76	306	5275	A	NT:H30
1-182-04	Yes	< 1 yr	M	no	7/4/09	6-month	S4	C1	1_182_04_S4_C1	JJLP00000000	SRS568244	5130349	50.74	109	2186	B1	O182:H23
1-182-04	Yes	< 1 yr	M	no	7/4/09	6-month	S4	C2	1_182_04_S4_C2	JJLM00000000	SRS568332	5151350	50.77	88	2186	B1	O182:H23
1-182-04	Yes	< 1 yr	M	no	7/4/09	6-month	S4	C3	1_182_04_S4_C3	JJLH00000000	SRS568031	5100380	50.76	106	2186	B1	O182:H23
1-250-04	Yes	< 1 yr	M	yes	1/12/09	baseline	S1	C1	1_250_04_S1_C1	JNQB00000000	SRS568022	5177057	50.62	108	449	D	O17/O44/O73/O77/O106:H18 Gp9
1-250-04	Yes	< 1 yr	M	yes	1/12/09	baseline	S1	C2	1_250_04_S1_C2	JNQC00000000	SRS568254	5159098	50.61	103	449	D	O17/O44/O73/O77/O106:H18 Gp9
1-250-04	Yes	< 1 yr	M	yes	1/12/09	baseline	S1	C3	1_250_04_S1_C3	JJLW00000000	SRS568159	5095530	50.59	169	449	D	O17/O44/O73/O77/O106:H18 Gp9
1-250-04	Yes	< 1 yr	M	no	4/11/09	3-month	S3	C1	1_250_04_S3_C1	JJLU00000000	SRS568284	5098119	50.93	203	3036	F	O97:H45
1-250-04	Yes	< 1 yr	M	no	4/11/09	3-month	S3	C2	1_250_04_S3_C2	JOSR00000000	SRS572002	5318327	50.91	306	3036	F	O97:H45
1-250-04	Yes	< 1 yr	M	no	7/4/09	6-month	S4	C1	1_250_04_S4_C1	JJLO00000000	SRS568193	5221656	50.84	162	746	A	O61:H21
1-250-04	Yes	< 1 yr	M	no	7/4/09	6-month	S4	C2	1_250_04_S4_C2	JJLL00000000	SRS568392	5235472	50.84	168	746	A	O61:H21
1-392-07	Yes	< 1 yr	M	no	1/12/09	baseline	S1	C1	1_392_07_S1_C1	JNPT00000000	SRS568024	5422993	50.42	174	38	D	O86:H5
1-392-07	Yes	< 1 yr	M	no	1/12/09	baseline	S1	C2	1_392_07_S1_C2	JNPU00000000	SRS568304	5490294	50.43	138	38	D	O86:H5
1-392-07	Yes	< 1 yr	M	no	4/9/09	3-month	S3	C1	1_392_07_S3_C1	JNPY00000000	SRS568368	4905892	50.91	218	1602	A	NT
1-392-07	Yes	< 1 yr	M	no	4/9/09	3-month	S3	C2	1_392_07_S3_C2	JNPP00000000	SRS568155	4906646	50.9	216	1602	A	NT
1-392-07	Yes	< 1 yr	M	no	4/9/09	3-month	S3	C3	1_392_07_S3_C3	JJLR00000000	SRS568326	4922974	50.9	222	1602	A	NT
1-392-07	Yes	< 1 yr	M	no	7/3/09	6-month	S4	C1	1_392_07_S4_C1	JOSK00000000	SRS572003	5805306	50.61	412	223	B1	O132:H16
1-392-07	Yes	< 1 yr	M	no	7/3/09	6-month	S4	C2	1_392_07_S4_C2	JJLK00000000	SRS568345	5052082	50.8	91	3497	B1	O8:H30
1-392-07	Yes	< 1 yr	M	no	7/3/09	6-month	S4	C3	1_392_07_S4_C3	JOSH00000000	SRS572004	5806384	50.62	268	223	B1	O132:H16
2-005-03	No	< 1 yr	M	yes	1/23/09	baseline	S1	C1	2_005_03_S1_C1	JJME00000000	SRS568279	5253303	50.56	154	5257	B1	O8:H12
2-005-03	No	< 1 yr	M	yes	1/23/09	baseline	S1	C2	2_005_03_S1_C2	JJMC00000000	SRS568145	5258795	50.54	155	5257	B1	O8:H12
2-005-03	No	< 1 yr	M	yes	1/23/09	baseline	S1	C3	2_005_03_S1_C3	JJLZ00000000	SRS568165	5271896	50.56	166	5257	E	O8:H12
2-005-03	No	< 1 yr	M	no	4/23/09	3-month	S3	C1	2_005_03_S3_C1	JNPQ00000000	SRS568327	5299384	50.67	168	2178	B1	O81:H27
2-005-03	No	< 1 yr	M	no	4/23/09	3-month	S3	C2	2_005_03_S3_C2	JMGT00000000	SRS568080	5310180	50.64	121	69	D	:O17/O44/O73/O77/O106:H18 Gp9
2-005-03	No	< 1 yr	M	no	4/23/09	3-month	S3	C3	2_005_03_S3_C3	JNPR00000000	SRS568354	5337905	50.63	168	2178	B1	O81:H27
2-005-03	No	< 1 yr	M	no	7/15/09	6-month	S4	C1	2_005_03_S4_C1	JNPS00000000	SRS568134	5266830	50.72	235	43	A	NT:H10
2-005-03	No	< 1 yr	M	no	7/15/09	6-month	S4	C2	2_005_03_S4_C2	JJLN00000000	SRS568239	5200769	50.68	284	452	B2	O81:H27
2-005-03	No	< 1 yr	M	no	7/15/09	6-month	S4	C3	2_005_03_S4_C3	JJLJ00000000	SRS568341	5246595	50.71	241	452	B2	O81:H27
2-011-08	No	2 yrs	F	no	1/23/09	baseline	S1	C1	2_011_08_S1_C1	JMGQ00000000	SRS568305	5331782	50.21	125	485	cryptic	Onovel3:H45
2-011-08	No	2 yrs	F	no	1/23/09	baseline	S1	C2	2_011_08_S1_C2	JJMB00000000	SRS568306	5278747	50.64	146	3018	E	NT:H41
2-011-08	No	2 yrs	F	no	1/23/09	baseline	S1	C3	2_011_08_S1_C3	JNNA00000000	SRS568205	4965207	50.89	159	165	A	NT:H2
2-011-08	No	2 yrs	F	no	4/23/09	3-month	S3	C1	2_011_08_S3_C1	JNNB00000000	SRS568026	5163084	50.68	169	10	A	O128ab/ac:H10
2-011-08	No	2 yrs	F	no	4/23/09	3-month	S3	C2	2_011_08_S3_C2	JMGU00000000	SRS568369	5301316	50.7	264	43	A	NT:H10
2-011-08	No	2 yrs	F	no	4/23/09	3-month	S3	C3	2_011_08_S3_C3	JMGV00000000	SRS568194	5146593	50.65	162	10	A	O128ab/ac:H10
2-011-08	No	2 yrs	F	no	7/15/09	6-month	S4	C1	2_011_08_S4_C1	JNNC00000000	SRS568360	4839184	50.91	183	216	A	O3:H4
2-011-08	No	2 yrs	F	no	7/15/09	6-month	S4	C3	2_011_08_S4_C3	JNND00000000	SRS568021	4815027	50.95	173	216	A	O3:H4
2-052-05	No	1 yr	F	yes	1/23/09	baseline	S1	C1	2_052_05_S1_C1	JMGR00000000	SRS568241	5270362	50.57	162	5257	B1	O8:H12
2-052-05	No	1 yr	F	yes	1/23/09	baseline	S1	C3	2_052_05_S1_C3	JNNE00000000	SRS568275	5231155	50.57	148	1286	B1	O71:H32
2-052-05	No	1 yr	F	no	4/23/09	3-month	S3	C1	2_052_05_S3_C1	JNNF00000000	SRS568199	4955132	50.64	135	1286	A	O71:H32

Subject and Isolate Data									Genome assembly information						Strain information		
Subject ID	MDA	Age	Sex	Diarrhea	Date	Time point	Time point ID	Colony	Assembly ID	WGS Accession	SRA Accession	Genome size	%GC	No. of contigs	MLST	Phylogroup	Serotype
2-052-05	No	1 yr	F	no	4/23/09	3-month	S3	C2	2_052_05_S3_C2	JOSJ00000000	SRS572007	5018629	50.64	100	1286	A	O71:H32
2-052-05	No	1 yr	F	no	4/23/09	3-month	S3	C3	2_052_05_S3_C3	JNOZ00000000	SRS568039	4926400	50.62	117	1286	A	O71:H32
2-052-05	No	1 yr	F	no	7/15/09	6-month	S4	C1	2_052_05_S4_C1	JNNG00000000	SRS568206	5304552	50.74	179	452	B2	O81:H7
2-052-05	No	1 yr	F	no	7/15/09	6-month	S4	C2	2_052_05_S4_C2	JNPA00000000	SRS568325	5287681	50.56	105	1891	B1	O121:H7
2-052-05	No	1 yr	F	no	7/15/09	6-month	S4	C3	2_052_05_S4_C3	JNPB00000000	SRS568355	4936995	50.91	155	10	A	O69:H32
2-156-04	No	2 yrs	M	no	1/23/09	baseline	S1	C3	2_156_04_S1_C3	JNPE00000000	SRS568394	4967237	50.87	189	5279	A	O90/O127:H21/H36 Gp 4
2-156-04	No	2 yrs	M	no	4/23/09	3-month	S3	C1	2_156_04_S3_C1	JNPF00000000	SRS568227	4986885	50.48	98	469	B1	O32:H16
2-156-04	No	2 yrs	M	no	4/23/09	3-month	S3	C2	2_156_04_S3_C2	JNPK00000000	SRS568285	4981016	50.33	87	3910	A	O8/O153var2:H33
2-156-04	No	2 yrs	M	no	4/23/09	3-month	S3	C3	2_156_04_S3_C3	JNPG00000000	SRS568370	5239835	50.66	130	165	A	O80:H19
2-156-04	No	2 yrs	M	no	7/15/09	6-month	S4	C1	2_156_04_S4_C1	JNPJ00000000	SRS568225	4901577	51.03	164	216	A	O36/Onovel3:H11/H34/H35
2-156-04	No	2 yrs	M	no	7/15/09	6-month	S4	C2	2_156_04_S4_C2	JNQD00000000	SRS568393	5095777	50.89	215	401	B1	O36:H11/H35
2-156-04	No	2 yrs	M	no	7/15/09	6-month	S4	C3	2_156_04_S4_C3	JNQE00000000	SRS568366	4911937	50.91	183	216	A	O154:H4/H55
2-177-06	No	1 yr	F	no	1/24/09	baseline	S1	C1	2_177_06_S1_C1	JNPH00000000	SRS568192	5236669	50.56	172	5257	B1	O8:H12
2-177-06	No	1 yr	F	no	1/24/09	baseline	S1	C2	2_177_06_S1_C2	JNPL00000000	SRS568319	5292127	50.5	145	5257	B1	O8:H12
2-177-06	No	1 yr	F	no	1/24/09	baseline	S1	C3	2_177_06_S1_C3	JNPM00000000	SRS568273	5232863	50.54	146	5257	B1	O8:H12
2-177-06	No	1 yr	F	yes	4/24/09	3-month	S3	C1	2_177_06_S3_C1	JNPI00000000	SRS568342	5153872	50.69	145	641	B1	Onovel20:H10
2-177-06	No	1 yr	F	yes	4/24/09	3-month	S3	C2	2_177_06_S3_C2	JNQF00000000	SRS568137	5360304	50.59	181	5386	A	O8/O179:H10
2-177-06	No	1 yr	F	yes	4/24/09	3-month	S3	C3	2_177_06_S3_C3	JOSO00000000	SRS572008	5301549	50.59	167	5386	A	O8/O179:H10
2-177-06	No	1 yr	F	no	7/16/09	6-month	S4	C1	2_177_06_S4_C1	JNQG00000000	SRS568347	5144646	50.85	102	117	B2	O161:H4
2-177-06	No	1 yr	F	no	7/16/09	6-month	S4	C2	2_177_06_S4_C2	JNPN00000000	SRS568078	5060232	50.86	113	2522	B1	O153var1:H12
2-177-06	No	1 yr	F	no	7/16/09	6-month	S4	C3	2_177_06_S4_C3	JNPZ00000000	SRS568356	5570876	50.63	191	678	B1	O104:H4
2-210-07	No	2 yrs	M	no	1/23/09	baseline	S1	C2	2_210_07_S1_C2	JNPV00000000	SRS568228	5155557	50.79	198	1415	A	O185:NT
2-210-07	No	2 yrs	M	no	1/23/09	baseline	S1	C3	2_210_07_S1_C3	JNPO00000000	SRS568136	5214871	50.88	169	1415	A	O185:NT
2-210-07	No	2 yrs	M	no	4/23/09	3-month	S3	C1	2_210_07_S3_C1	JNQH00000000	SRS568395	4709037	50.84	115	744	A	O89:H9
2-210-07	No	2 yrs	M	no	4/23/09	3-month	S3	C2	2_210_07_S3_C2	JNPX00000000	SRS568061	5169282	50.74	243	40	B1	O112ab:H21
2-210-07	No	2 yrs	M	no	4/23/09	3-month	S3	C3	2_210_07_S3_C3	JNQA00000000	SRS568337	5394252	50.53	341	378	A	O89/O101/O162:H33 Gp15
2-210-07	No	2 yrs	M	no	7/15/09	6-month	S4	C1	2_210_07_S4_C1	JNPW00000000	SRS568378	4974306	50.43	85	410	B1	O8:H9
2-210-07	No	2 yrs	M	no	7/15/09	6-month	S4	C2	2_210_07_S4_C2	JNQI00000000	SRS568283	4923516	50.93	187	216	A	O154:H4/H55
2-210-07	No	2 yrs	M	no	7/15/09	6-month	S4	C3	2_210_07_S4_C3	JNQJ00000000	SRS568079	4911172	50.93	185	216	A	O154:H4/H55
2-222-05	No	< 1 yr	F	no	1/23/09	baseline	S1	C1	2_222_05_S1_C1	JNQK00000000	SRS568151	4965006	50.53	108	5376	B1	O39:H12
2-222-05	No	< 1 yr	F	no	1/23/09	baseline	S1	C2	2_222_05_S1_C2	JNQL00000000	SRS568246	4931696	50.56	106	5376	B1	O39:H12
2-222-05	No	< 1 yr	F	no	1/23/09	baseline	S1	C3	2_222_05_S1_C3	JNQM00000000	SRS568320	4943872	50.55	105	5376	B1	O39:H12
2-222-05	No	< 1 yr	F	yes	4/23/09	3-month	S3	C1	2_222_05_S3_C1	JOSM00000000	SRS572010	5479430	50.74	269	10	A	O15:H4
2-222-05	No	< 1 yr	F	yes	4/23/09	3-month	S3	C2	2_222_05_S3_C2	JOSN00000000	SRS572012	5388923	50.78	245	10	A	O15:H4
2-222-05	No	< 1 yr	F	yes	4/23/09	3-month	S3	C3	2_222_05_S3_C3	JNQN00000000	SRS568029	5471232	50.74	211	10	A	O15:H4
2-222-05	No	< 1 yr	F	no	7/15/09	6-month	S4	C1	2_222_05_S4_C1	JORR00000000	SRS568281	4862082	50.95	161	216	A	O154:H4/H55
2-222-05	No	< 1 yr	F	no	7/15/09	6-month	S4	C2	2_222_05_S4_C2	JNQO00000000	SRS568286	5257015	50.4	174	484	A	O7:H4
2-222-05	No	< 1 yr	F	no	7/15/09	6-month	S4	C3	2_222_05_S4_C3	JOSP00000000	SRS572028	5239725	50.61	200	484	A	O7:H4
2-316-03	No	< 1 yr	F	no	1/23/09	baseline	S1	C1	2_316_03_S1_C1	JOMW00000000	SRS572029	5396154	50.68	196	69	B2	O17/O44/O73/O77/O106:H18 Gp9
2-316-03	No	< 1 yr	F	no	1/23/09	baseline	S1	C2	2_316_03_S1_C2	JOMX00000000	SRS572032	5341596	50.65	162	69	B2	O17/O44/O73/O77/O106:H18 Gp9
2-316-03	No	< 1 yr	F	yes	4/24/09	3-month	S3	C1	2_316_03_S3_C1	JNQP00000000	SRS568274	5075779	50.97	151	761	A	O21:H25
2-316-03	No	< 1 yr	F	yes	4/24/09	3-month	S3	C2	2_316_03_S3_C2	JNQQ00000000	SRS568153	4983084	50.76	116	155	B1	O9/Onovel14:H21
2-316-03	No	< 1 yr	F	yes	4/24/09	3-month	S3	C3	2_316_03_S3_C3	JNQR00000000	SRS568271	4815859	50.8	119	155	B1	O9:H21
2-316-03	No	< 1 yr	F	no	7/15/09	6-month	S4	C1	2_316_03_S4_C1	JNQS00000000	SRS568338	5226094	50.57	154	394	B2	O17/O44/O73/O77/O106:H18 Gp9
2-316-03	No	< 1 yr	F	no	7/15/09	6-month	S4	C2	2_316_03_S4_C2	JNQT00000000	SRS568207	5332986	50.62	210	10	A	O107/O117:H10 Gp8
2-316-03	No	< 1 yr	F	no	7/15/09	6-month	S4	C3	2_316_03_S4_C3	JNQU00000000	SRS568146	5427639	50.68	155	131	B2	O18ab/ac:H4 Gp12
2-427-07	No	2 yrs	M	no	1/23/09	baseline	S1	C1	2_427_07_S1_C1	JNQV00000000	SRS568255	4710651	50.66	139	155	B1	O9/O112ac:H51
2-427-07	No	2 yrs	M	no	1/23/09	baseline	S1	C2	2_427_07_S1_C2	JNQW00000000	SRS568160	4796164	50.6	135	155	B1	O9/O112ac:H21/H51
2-427-07	No	2 yrs	M	no	1/23/09	baseline	S1	C3	2_427_07_S1_C3	JNRJ00000000	SRS568190	5196316	50.69	145	155	B1	O9/O103/O112ac:H16/H21
2-427-07	No	2 yrs	M	no	4/23/09	3-month	S3	C1	2_427_07_S3_C1	JNQX00000000	SRS568157	5287373	50.73	120	2175	B1	O103:H16
2-427-07	No	2 yrs	M	no	4/23/09	3-month	S3	C3	2_427_07_S3_C3	JNQY00000000	SRS568162	4875912	50.7	88	2175	B1	O103:H16
2-427-07	No	2 yrs	M	no	7/15/09	6-month	S4	C1	2_427_07_S4_C1	JNQZ00000000	SRS568339	5248071	50.66	194	382	A	NT:H5
2-427-07	No	2 yrs	M	no	7/15/09	6-month	S4	C2	2_427_07_S4_C2	JNRA00000000	SRS568141	5560615	50.71	141	131	B2	O25/Onovel31:H4
2-427-07	No	2 yrs	M	no	7/15/09	6-month	S4	C3	2_427_07_S4_C3	JOMY00000000	SRS572033	5732927	50.57	227	223	B1	O132:H16
2-460-02	No	1 yr	M	yes	1/23/09	baseline	S1	C1	2_460_02_S1_C1	JOSS00000000	SRS572036	5607136	50.6	197	223	B1	O132:H16
2-460-02	No	1 yr	M	yes	1/23/09	baseline	S1	C2	2_460_02_S1_C2	JOSU00000000	SRS572037	5659114	50.73	172	131	B2	O25/O31:H4
2-460-02	No	1 yr	M	yes	1/23/09	baseline	S1	C3	2_460_02_S1_C3	JOMZ00000000	SRS572038	5544292	50.69	123	131	B2	O25/Onovel31:H4
2-460-02	No	1 yr	M	no	4/23/09	3-month	S3	C1	2_460_02_S3_C1	JNRB00000000	SRS568278	5475690	50.79	250	3281	A	O111:H12
2-460-02	No	1 yr	M	no	4/23/09	3-month	S3	C2	2_460_02_S3_C2	JNRC00000000	SRS568261	5465378	50.76	293	3281	A	O111:H12
2-460-02	No	1 yr	M	no	4/23/09	3-month	S3	C3	2_460_02_S3_C3	JNRD00000000	SRS568077	5367092	50.91	299	3281	A	O111:H12
2-460-02	No	1 yr	M	no	7/15/09	6-month	S4	C1	2_460_02_S4_C1	JONA00000000	SRS572039	5541966	50.64	174	43	A	O6:H10
2-460-02	No	1 yr	M	no	7/15/09	6-month	S4	C2	2_460_02_S4_C2	JNRE00000000	SRS568371	5346503	50.54	175	43	A	O6:H10

Subject and Isolate Data									Genome assembly information						Strain information		
Subject ID	MDA	Age	Sex	Diarrhea	Date	Time point	Time point ID	Colony	Assembly ID	WGS Accession	SRA Accession	Genome size	%GC	No. of contigs	MLST	Phylogroup	Serotype
2-460-02	No	1 yr	M	no	7/15/09	6-month	S4	C3	2_460_02_S4_C3	JNRF00000000	SRS568391	5345933	50.55	172	43	A	O6:H10
2-474-04	No	2 yrs	M	no	1/24/09	baseline	S1	C1	2_474_04_S1_C1	JNRG00000000	SRS568268	5124973	50.91	201	1415	A	O76:H34
2-474-04	No	2 yrs	M	no	1/24/09	baseline	S1	C2	2_474_04_S1_C2	JNRK00000000	SRS568170	4925970	50.85	143	226	A	O85:H10
2-474-04	No	2 yrs	M	no	4/24/09	3-month	S3	C1	2_474_04_S3_C1	JNRH00000000	SRS568280	5446772	50.68	333	43	A	NT:H10
2-474-04	No	2 yrs	M	no	4/24/09	3-month	S3	C2	2_474_04_S3_C2	JNRI00000000	SRS568358	5362630	50.7	248	43	A	NT:H10
2-474-04	No	2 yrs	M	no	4/24/09	3-month	S3	C3	2_474_04_S3_C3	JNRL00000000	SRS568377	5448064	50.7	308	43	A	NT:H10
2-474-04	No	2 yrs	M	no	7/16/09	6-month	S4	C1	2_474_04_S4_C1	JOSL00000000	SRS568075	5039270	50.8	94	1125	B1	O139:H19
2-474-04	No	2 yrs	M	no	7/16/09	6-month	S4	C2	2_474_04_S4_C2	JNRM00000000	SRS568361	5044800	50.77	87	1125	B1	O139:H19
2-474-04	No	2 yrs	M	no	7/16/09	6-month	S4	C3	2_474_04_S4_C3	JNRN00000000	SRS568373	5028064	50.78	93	1125	B1	O139:H19
3-020-07	Yes	< 1 yr	F	yes	1/16/09	baseline	S1	C1	3_020_07_S1_C1	JNRO00000000	SRS568135	5091775	50.82	177	1139	A	O8:H9
3-020-07	Yes	< 1 yr	F	yes	1/16/09	baseline	S1	C2	3_020_07_S1_C2	JNRP00000000	SRS568201	5032286	50.83	121	1139	A	O8:H9
3-020-07	Yes	< 1 yr	F	yes	1/16/09	baseline	S1	C3	3_020_07_S1_C3	JNRQ00000000	SRS568390	5070287	50.78	155	1139	A	O8:H9
3-020-07	Yes	< 1 yr	F	no	4/16/09	3-month	S3	C1	3_020_07_S3_C1	JNRR00000000	SRS568049	5514688	50.53	192	43	A	O6:H10
3-020-07	Yes	< 1 yr	F	no	4/16/09	3-month	S3	C2	3_020_07_S3_C2	JONB00000000	SRS572096	5633558	50.57	250	43	A	O6:H10
3-020-07	Yes	< 1 yr	F	no	7/8/09	6-month	S4	C1	3_020_07_S4_C1	JONC00000000	SRS572099	5691481	50.64	129	131	B2	O18ab/ac:H4 Gp12
3-020-07	Yes	< 1 yr	F	no	7/8/09	6-month	S4	C2	3_020_07_S4_C2	JNRS00000000	SRS568247	5260928	50.34	191	5617	B1	O131:H8
3-020-07	Yes	< 1 yr	F	no	7/8/09	6-month	S4	C3	3_020_07_S4_C3	JNRT00000000	SRS568235	5298396	50.38	162	5617	B1	O131:H8
3-073-06	Yes	2 yrs	M	no	1/17/09	baseline	S1	C1	3_073_06_S1_C1	JNRU00000000	SRS568208	4945389	50.56	82	4554	B2	O157:H39
3-073-06	Yes	2 yrs	M	no	1/17/09	baseline	S1	C2	3_073_06_S1_C2	JNRV00000000	SRS568272	5248248	50.93	274	10	A	O29:H4
3-073-06	Yes	2 yrs	M	no	4/17/09	3-month	S3	C1	3_073_06_S3_C1	JNRW00000000	SRS568163	5180914	50.77	223	43	A	NT:H10
3-073-06	Yes	2 yrs	M	no	4/17/09	3-month	S3	C2	3_073_06_S3_C2	JNRX00000000	SRS568372	5256796	50.64	226	43	A	NT:H10
3-073-06	Yes	2 yrs	M	no	7/9/09	6-month	S4	C1	3_073_06_S4_C1	JNMT00000000	SRS568203	5408383	50.79	252	1178	A	O130:H26
3-073-06	Yes	2 yrs	M	no	7/9/09	6-month	S4	C2	3_073_06_S4_C2	JNRY00000000	SRS568276	4831902	50.83	112	5474	B1	Onovel4:H10
3-073-06	Yes	2 yrs	M	no	7/9/09	6-month	S4	C3	3_073_06_S4_C3	JNRZ00000000	SRS568007	5361656	50.81	226	1178	A	O130:H26
3-105-05	Yes	1 yr	F	yes	1/17/09	baseline	S1	C1	3_105_05_S1_C1	JNLZ00000000	SRS568322	5152314	50.99	188	757	A	NT:H20
3-105-05	Yes	1 yr	F	yes	1/17/09	baseline	S1	C2	3_105_05_S1_C2	JNSA00000000	SRS568232	5048264	50.98	231	206	A	O60:H5
3-105-05	Yes	1 yr	F	yes	1/17/09	baseline	S1	C3	3_105_05_S1_C3	JNSB00000000	SRS568256	5239307	51.04	187	757	A	NT:H20
3-105-05	Yes	1 yr	F	no	4/17/09	3-month	S3	C1	3_105_05_S3_C1	JNMA00000000	SRS568307	5117966	50.54	182	10	A	O78:H2
3-105-05	Yes	1 yr	F	no	4/17/09	3-month	S3	C2	3_105_05_S3_C2	JNMB00000000	SRS568166	5097926	50.56	179	10	A	O78:H2
3-105-05	Yes	1 yr	F	no	4/17/09	3-month	S3	C3	3_105_05_S3_C3	JNMO00000000	SRS568321	4995834	50.81	200	2172	A	O9:H4
3-105-05	Yes	1 yr	F	no	7/9/09	6-month	S4	C1	3_105_05_S4_C1	JNML00000000	SRS568365	4929019	50.81	85	227	A	O89/O101/O162:H10 Gp15
3-105-05	Yes	1 yr	F	no	7/9/09	6-month	S4	C2	3_105_05_S4_C2	JNMC00000000	SRS568059	5353813	50.4	116	12	B2	O18ab/ac:H5
3-105-05	Yes	1 yr	F	no	7/9/09	6-month	S4	C3	3_105_05_S4_C3	JNMD00000000	SRS568389	5098282	50.94	242	1114	A	O35:H26
3-267-03	Yes	1 yr	F	yes	1/16/09	baseline	S1	C1	3_267_03_S1_C1	JNMP00000000	SRS568324	5453130	50.74	236	5251	B1	O128:H8
3-267-03	Yes	1 yr	F	yes	1/16/09	baseline	S1	C2	3_267_03_S1_C2	JNMN00000000	SRS568375	5115347	50.54	87	3056	D	O51:H42
3-267-03	Yes	1 yr	F	yes	1/16/09	baseline	S1	C3	3_267_03_S1_C3	JNME00000000	SRS568030	5313998	50.71	219	5251	B1	O128:H8
3-267-03	Yes	1 yr	F	no	4/16/09	3-month	S3	C1	3_267_03_S3_C1	JNMF00000000	SRS568381	5133769	50.79	180	10	A	NT:H16
3-267-03	Yes	1 yr	F	no	4/16/09	3-month	S3	C2	3_267_03_S3_C2	JNMM00000000	SRS568204	4850588	50.71	79	101	B1	O153var1:H2
3-267-03	Yes	1 yr	F	no	7/8/09	6-month	S4	C1	3_267_03_S4_C1	JOND00000000	SRS572276	5647831	50.63	238	223	B1	O132:H16
3-267-03	Yes	1 yr	F	no	7/8/09	6-month	S4	C2	3_267_03_S4_C2	JNMR00000000	SRS568333	5390559	50.5	185	5285	B1	O163:H19
3-373-03	Yes	< 1 yr	F	no	1/16/09	baseline	S1	C1	3_373_03_S1_C1	JNMV00000000	SRS568250	5089359	50.84	152	5293	A	O60:H5
3-373-03	Yes	< 1 yr	F	no	1/16/09	baseline	S1	C2	3_373_03_S1_C2	JNMI00000000	SRS568252	5066961	50.8	178	5293	A	O60:H5
3-373-03	Yes	< 1 yr	F	no	1/16/09	baseline	S1	C3	3_373_03_S1_C3	JNMU00000000	SRS568251	5131811	50.88	171	5293	A	O60:H5
3-373-03	Yes	< 1 yr	F	no	4/16/09	3-month	S3	C1	3_373_03_S3_C1	JNMG00000000	SRS568349	4627215	50.77	117	48	A	O9:H11
3-373-03	Yes	< 1 yr	F	no	4/16/09	3-month	S3	C2	3_373_03_S3_C2	JNMQ00000000	SRS568242	4808501	50.78	82	10	A	O10:H40
3-373-03	Yes	< 1 yr	F	no	4/16/09	3-month	S3	C3	3_373_03_S3_C3	JNMH00000000	SRS568143	4871839	50.8	107	10	A	O10:H40
3-373-03	Yes	< 1 yr	F	no	7/8/09	6-month	S4	C1	3_373_03_S4_C1	JNMW00000000	SRS568169	5294790	50.62	171	10	A	O16:H48
3-373-03	Yes	< 1 yr	F	no	7/8/09	6-month	S4	C2	3_373_03_S4_C2	JNMS00000000	SRS568020	5328850	50.58	203	10	A	O16:H48
3-373-03	Yes	< 1 yr	F	no	7/8/09	6-month	S4	C3	3_373_03_S4_C3	JOQL00000000	SRS568359	5294474	50.61	180	10	A	O16:H48
3-475-03	Yes	1 yr	M	no	1/16/09	baseline	S1	C1	3_475_03_S1_C1	JNMJ00000000	SRS568270	4789923	50.87	194	5372	A	O90/O127:H4 Gp 4
3-475-03	Yes	1 yr	M	no	1/16/09	baseline	S1	C2	3_475_03_S1_C2	JOQC00000000	SRS568289	5512344	50.62	176	200	B1	O127:H27
3-475-03	Yes	1 yr	M	no	4/16/09	3-month	S3	C1	3_475_03_S3_C1	JOQD00000000	SRS568234	5275184	50.64	145	328	B1	O88:H25
3-475-03	Yes	1 yr	M	no	4/16/09	3-month	S3	C2	3_475_03_S3_C2	JOQE00000000	SRS568243	5448404	50.69	203	29	B1	O71:H8
3-475-03	Yes	1 yr	M	yes	7/8/09	6-month	S4	C1	3_475_03_S4_C1	JNMK00000000	SRS568330	4929218	50.66	105	10	A	NT:H10
3-475-03	Yes	1 yr	M	yes	7/8/09	6-month	S4	C2	3_475_03_S4_C2	JNMX00000000	SRS568302	5055047	51	170	757	A	NT:H20
4-203-08	Yes	1 yr	M	no	1/19/09	baseline	S1	C1	4_203_08_S1_C1	JNMY00000000	SRS568036	5270688	50.75	173	10	A	O107/O117:H10 Gp8
4-203-08	Yes	1 yr	M	no	1/19/09	baseline	S1	C2	4_203_08_S1_C2	JOQF00000000	SRS568245	5325397	50.76	183	10	A	O107/O117:H10 Gp8
4-203-08	Yes	1 yr	M	no	1/19/09	baseline	S1	C3	4_203_08_S1_C3	JOQG00000000	SRS568253	5302353	50.74	167	10	A	O107/O117:H10 Gp8
4-203-08	Yes	1 yr	M	no	4/18/09	3-month	S3	C1	4_203_08_S3_C1	JOQJ00000000	SRS568060	5030989	50.53	171	5318	A	NT:H10
4-203-08	Yes	1 yr	M	no	4/18/09	3-month	S3	C2	4_203_08_S3_C2	JOQK00000000	SRS568269	4760420	50.79	105	5518	A	O38:H26
4-203-08	Yes	1 yr	M	no	4/18/09	3-month	S3	C3	4_203_08_S3_C3	JOQH00000000	SRS568237	4777481	50.78	116	5518	A	O38:H26
4-203-08	Yes	1 yr	M	no	7/10/09	6-month	S4	C2	4_203_08_S4_C2	JOQI00000000	SRS568195	5069922	50.6	172	5318	A	NT:H10

Subject and Isolate Data									Genome assembly information						Strain information		
Subject ID	MDA	Age	Sex	Diarrhea	Date	Time point	Time point ID	Colony	Assembly ID	WGS Accession	SRA Accession	Genome size	%GC	No. of contigs	MLST	Phylogroup	Serotype
4-203-08	Yes	1 yr	M	no	7/10/09	6-month	S4	C3	4_203_08_S4_C3	JNMZ00000000	SRS568265	5007529	50.56	180	5318	A	NT:H10
5-172-05	No	< 1 yr	M	yes	1/22/09	baseline	S1	C3	5_172_05_S1_C3	JOQS00000000	SRS568171	4853958	51.02	185	1421	A	NT:H45
5-172-05	No	< 1 yr	M	no	4/22/09	3-month	S3	C1	5_172_05_S3_C1	JOQQ00000000	SRS568363	5089646	50.59	181	5439	A	NT:H10
5-172-05	No	< 1 yr	M	no	4/22/09	3-month	S3	C3	5_172_05_S3_C3	JOQO00000000	SRS568364	5192502	50.79	193	206	A	O60:H5
5-172-05	No	< 1 yr	M	yes	7/14/09	6-month	S4	C1	5_172_05_S4_C1	JOQP00000000	SRS568287	5046870	50.48	113	10	A	O99:H33
5-172-05	No	< 1 yr	M	yes	7/14/09	6-month	S4	C2	5_172_05_S4_C2	JOQN00000000	SRS568353	5060331	50.47	109	10	A	O99:H33
5-172-05	No	< 1 yr	M	yes	7/14/09	6-month	S4	C3	5_172_05_S4_C3	JOQT00000000	SRS568323	5012574	50.5	111	10	A	O99:H33
5-366-08	No	< 1 yr	F	no	1/22/09	baseline	S1	C1	5_366_08_S1_C1	JOQU00000000	SRS568240	5446335	50.59	123	2914	D	O166:H15
5-366-08	No	< 1 yr	F	no	1/22/09	baseline	S1	C3	5_366_08_S1_C3	JONE00000000	SRS572100	5440914	50.64	109	2914	D	O166:H15
5-366-08	No	< 1 yr	F	no	4/22/09	3-month	S3	C1	5_366_08_S3_C1	JOQY00000000	SRS568226	5331786	50.68	124	2	D	O17/O44/O73/O77/O106:H18 Gp9
5-366-08	No	< 1 yr	F	no	4/22/09	3-month	S3	C2	5_366_08_S3_C2	JOQW00000000	SRS568266	5404125	50.52	174	5285	B1	O163:H19
5-366-08	No	< 1 yr	F	no	4/22/09	3-month	S3	C3	5_366_08_S3_C3	JOQV00000000	SRS568138	5254175	50.71	109	2	D	O17/O44/O73/O77/O106:H18 Gp9
5-366-08	No	< 1 yr	F	no	7/14/09	6-month	S4	C1	5_366_08_S4_C1	JOST00000000	SRS572277	5645469	50.36	217	5793	A	O176:H33
5-366-08	No	< 1 yr	F	no	7/14/09	6-month	S4	C2	5_366_08_S4_C2	JOQM00000000	SRS568238	5215424	50.55	225	5348	A	O6:O16
6-175-07	Yes	1 yr	M	yes	1/15/09	baseline	S1	C1	6_175_07_S1_C1	JOQR00000000	SRS568288	4976991	50.69	102	583	B2	O63:H6
6-175-07	Yes	1 yr	M	yes	1/15/09	baseline	S1	C2	6_175_07_S1_C2	JOMS00000000	SRS568233	5311695	50.53	146	38	D	O153var1:H2
6-175-07	Yes	1 yr	M	yes	1/15/09	baseline	S1	C3	6_175_07_S1_C3	JORL00000000	SRS568376	5362266	50.58	127	38	D	O153var1:H2
6-175-07	Yes	1 yr	M	no	4/15/09	3-month	S3	C1	6_175_07_S3_C1	JOQZ00000000	SRS568231	5237667	50.56	106	827	B2	O4:H40
6-175-07	Yes	1 yr	M	no	4/15/09	3-month	S3	C2	6_175_07_S3_C2	JONF00000000	SRS572278	5672404	50.51	124	827	B2	O4:H40
6-175-07	Yes	1 yr	M	no	4/15/09	3-month	S3	C3	6_175_07_S3_C3	JORA00000000	SRS568189	5370013	50.51	106	827	B2	O4:H40
6-175-07	Yes	1 yr	M	no	7/7/09	6-month	S4	C1	6_175_07_S4_C1	JORB00000000	SRS568236	4778266	50.81	125	5518	A	O38:H26
6-175-07	Yes	1 yr	M	no	7/7/09	6-month	S4	C2	6_175_07_S4_C2	JORC00000000	SRS568380	4731158	50.77	103	5518	A	O38:H26
6-175-07	Yes	1 yr	M	no	7/7/09	6-month	S4	C3	6_175_07_S4_C3	JORK00000000	SRS568202	4811932	50.75	133	5518	A	O38:H26
6-319-05	Yes	< 1 yr	M	yes	1/15/09	baseline	S1	C1	6_319_05_S1_C1	JORT00000000	SRS568149	5228177	50.76	221	443	B1	O115:H5
6-319-05	Yes	< 1 yr	M	yes	1/15/09	baseline	S1	C2	6_319_05_S1_C2	JORD00000000	SRS568161	5166967	50.88	192	unknown	B1	O115:H5
6-319-05	Yes	< 1 yr	M	yes	1/15/09	baseline	S1	C3	6_319_05_S1_C3	JORE00000000	SRS568374	5338618	50.83	220	443	B1	O115:H5
6-319-05	Yes	< 1 yr	M	no	4/15/09	3-month	S3	C1	6_319_05_S3_C1	JORF00000000	SRS568346	5362601	50.48	113	827	B2	O4:H40
6-319-05	Yes	< 1 yr	M	no	4/15/09	3-month	S3	C2	6_319_05_S3_C2	JORG00000000	SRS568334	5363959	50.54	129	827	B2	O4:H40
6-319-05	Yes	< 1 yr	M	no	4/15/09	3-month	S3	C3	6_319_05_S3_C3	JORM00000000	SRS568257	5285772	50.57	103	827	B2	O4:H40
6-319-05	Yes	< 1 yr	M	no	7/7/09	6-month	S4	C2	6_319_05_S4_C2	JORH00000000	SRS568282	5066643	50.78	209	5294	A	O58:H32
6-319-05	Yes	< 1 yr	M	no	7/7/09	6-month	S4	C3	6_319_05_S4_C3	JORJ00000000	SRS568329	5209264	50.7	439	5294	A	O58:H32
6-537-08	Yes	1 yr	F	yes	1/15/09	baseline	S1	C1	6_537_08_S1_C1	JORI00000000	SRS568396	5286217	50.79	153	2332	A	O128ab/ac:H45
6-537-08	Yes	1 yr	F	yes	1/15/09	baseline	S1	C2	6_537_08_S1_C2	JOSC00000000	SRS568335	5310824	50.77	160	2332	A	O128ab/ac:H45
6-537-08	Yes	1 yr	F	yes	1/15/09	baseline	S1	C3	6_537_08_S1_C3	JOSF00000000	SRS568362	5325906	50.8	167	2332	A	O128ab/ac:H45
6-537-08	Yes	1 yr	F	no	4/15/09	3-month	S3	C1	6_537_08_S3_C1	JORP00000000	SRS568259	5168278	50.63	97	192	B1	O174:H7
6-537-08	Yes	1 yr	F	no	4/15/09	3-month	S3	C2	6_537_08_S3_C2	JORX00000000	SRS568154	5160700	50.56	98	192	B1	O174:H7
6-537-08	Yes	1 yr	F	no	4/15/09	3-month	S3	C3	6_537_08_S3_C3	JORQ00000000	SRS568303	5207327	50.57	99	192	B1	O174:H7
6-537-08	Yes	1 yr	F	no	7/7/09	6-month	S4	C1	6_537_08_S4_C1	JORS00000000	SRS568357	4806027	50.8	129	5518	A	O38:H26
6-537-08	Yes	1 yr	F	no	7/7/09	6-month	S4	C2	6_537_08_S4_C2	JOSG00000000	SRS568267	4789699	50.76	112	5518	A	O38:H26
7-233-03	No	1 yr	M	yes	1/27/09	baseline	S1	C2	7_233_03_S1_C2	JORN00000000	SRS568014	4763538	50.86	84	5334	A	O8/O32:H10
7-233-03	No	1 yr	M	yes	1/27/09	baseline	S1	C3	7_233_03_S1_C3	JORU00000000	SRS568398	4743985	50.84	72	5334	A	O8/O32:H10
7-233-03	No	1 yr	M	no	4/28/09	3-month	S3	C1	7_233_03_S3_C1	JORO00000000	SRS568316	5264612	50.69	140	10	A	O128:H10
7-233-03	No	1 yr	M	no	4/28/09	3-month	S3	C2	7_233_03_S3_C2	JORY00000000	SRS568147	5260199	50.84	204	1178	A	O130:H26
7-233-03	No	1 yr	M	no	4/28/09	3-month	S3	C3	7_233_03_S3_C3	JORV00000000	SRS568336	4962459	50.72	172	10	A	O128ab/ac:H10
7-233-03	No	1 yr	M	no	7/18/09	6-month	S4	C1	7_233_03_S4_C1	JOSD00000000	SRS568387	4673739	50.92	90	10	A	Onovel8:H32
7-233-03	No	1 yr	M	no	7/18/09	6-month	S4	C2	7_233_03_S4_C2	JORW00000000	SRS568277	4837509	50.61	83	4995	A	Onovel15:H16
7-233-03	No	1 yr	M	no	7/18/09	6-month	S4	C3	7_233_03_S4_C3	JOSE00000000	SRS568144	4641553	50.92	115	10	A	Onovel8:H32
8-415-05	No	< 1 yr	F	no	1/28/09	baseline	S1	C1	8_415_05_S1_C1	JORZ00000000	SRS568388	4819146	50.52	132	226	A	O8/O40:H4
8-415-05	No	< 1 yr	F	no	1/28/09	baseline	S1	C2	8_415_05_S1_C2	JOSQ00000000	SRS568350	4914937	50.48	155	226	A	O8/O40:H4
8-415-05	No	< 1 yr	F	no	4/30/09	3-month	S3	C1	8_415_05_S3_C1	JOSB00000000	SRS568317	5340365	50.46	154	73	B2	O6:H1/H12
8-415-05	No	< 1 yr	F	no	4/30/09	3-month	S3	C2	8_415_05_S3_C2	JOSI00000000	SRS568318	5290814	50.49	134	73	B2	O6:H1/H12
8-415-05	No	< 1 yr	F	no	4/30/09	3-month	S3	C3	8_415_05_S3_C3	JOSA00000000	SRS568290	5306490	50.46	152	73	B2	O6:H1/H12
8-415-05	No	< 1 yr	F	no	7/20/09	6-month	S4	C1	8_415_05_S4_C1	JOMT00000000	SRS568150	5275845	50.48	141	73	B2	O6:H1/H12
8-415-05	No	< 1 yr	F	no	7/20/09	6-month	S4	C2	8_415_05_S4_C2	JOMU00000000	SRS568258	5245147	50.48	128	unknown	B2	O6:H1/H12
8-415-05	No	< 1 yr	F	no	7/20/09	6-month	S4	C3	8_415_05_S4_C3	JOMV00000000	SRS568074	5329443	50.47	139	73	B2	O6:H1/H12
