# Supplementary material for: Temporal Variability of Escherichia coli Diversity in the Gastrointestinal Tracts of Tanzanian Children with and without Exposure to Antibiotics
Source: mSphere. 2018 Nov 7;3(6):e00558-18. doi: 10.1128/mSphere.00558-18 (PMC6222053; doi:10.1128/mSphere.00558-18)
Supplement: TABLE S2 [file sph006182690st2.pdf]

Table S2: Reference genomes and corresponding pathotypes

Tree label	Reference genomes	Pathotype/origi	Phylogroup	GenBank Accession
E. coli E110019	Escherichia coli E110019	EPEC	B1	NZ_AAJW00000000.2
E. coli E2348_69	Escherichia coli O127:H6 E2348/69	EPEC	B2	FM180568.1
E. coli E24377A	Escherichia coli E24377A	ETEC	B1	NC_009801.1
E. coli 53638	Escherichia coli 53638	EIEC	A	AAKB00000000.2
E. coli 536	Escherichia coli 536	UPEC	B2	NC_008253.1
E. coli 55989	Escherichia coli 55989	EAEC	B1	NC_011748.1
E. coli ATCC 8739	Escherichia coli ATCC 8739	lab adapted	A	NC_010468.1
E. coli B171	Escherichia coli B171	EPEC	B1	NZ_AAJX00000000.2
E. coli B7A	Escherichia coli B7A	ETEC	B1	NZ_CP005998.1
E. coli BL21	Escherichia coli BL21	lab adapted	A	NC_012947.1
E. coli BW2952	Escherichia coli BW2952	lab adapted	A	NC_012759.1
E. coli CFT073	Escherichia coli CFT073	UPEC	B2	AE014075.1
E. coli E22	Escherichia coli E22	EPEC	B1	NZ_AAJV00000000.2
E. coli H10407	Escherichia coli H10407	ETEC	A	NC_017633.1
E. coli HS	Escherichia coli HS	fecal isolate	A	NC_009800.1
E. coli IAI1	Escherichia coli IAI1	fecal isolate	B1	NC_011741.1
E. coli IAI39	Escherichia coli IAI39	ExPEC	F	NC_011750.1
E. coli 11368	Escherichia coli O26:H11 11368	EHEC	B1	NC_013361.1
E. coli O111 11128	Escherichia coli O111 11128	EHEC	B1	NC_013364.1
E. coli EDL933	Escherichia coli O157:H7 EDL933	EHEC	E	NC_002655.2
E. coli Sakai	Escherichia coli O157:H7 str Sakai	EHEC	E	NC_002695.1
E. coli 042	Escherichia coli O44:H18 042	EAEC	D	FN554766.1
E. coli CB9615	Escherichia coli O55:H7 CB9615	EPEC	E	NC_013941.1
E. coli S88	Escherichia coli O45:K1:H7 S88	ExPEC	B2	NC_011742.1
E. coli SE11	Escherichia coli O152:H28 SE11	fecal isolate	B1	NC_011415.1
E. coli SMS 3 5	Escherichia coli SMS 3-5	environmental	F	CP000970.1
E. coli TY 2482	Escherichia coli O104:H4 TY-2482	EHEC	B1	AFOG00000000.1
E. coli UMN026	Escherichia coli O17:K52:H18 UMN026	ExPEC	D	NC_011751.1
E. coli UTI89	Escherichia coli UTI89	UPEC	B2	NC_007946.1
S. boydii 3083	Shigella boydii 3083-94	Shigella	B1	NC_010658.1
S. dysenteriae	Shigella dysenteriae	Shigella	E	NC_007606.1
S. flexneri 2A	Shigella flexneri 2a	Shigella	B1	NC_004741.1
S. sonnei 046	Shigella sonnei 046	Shigella	B1	NC_007384.1
