# Supplementary figures and images for: Temporal Variability of Escherichia coli Diversity in the Gastrointestinal Tracts of Tanzanian Children with and without Exposure to Antibiotics
Source: mSphere. 2018 Nov 7;3(6):e00558-18. doi: 10.1128/mSphere.00558-18 (PMC6222053; doi:10.1128/mSphere.00558-18)

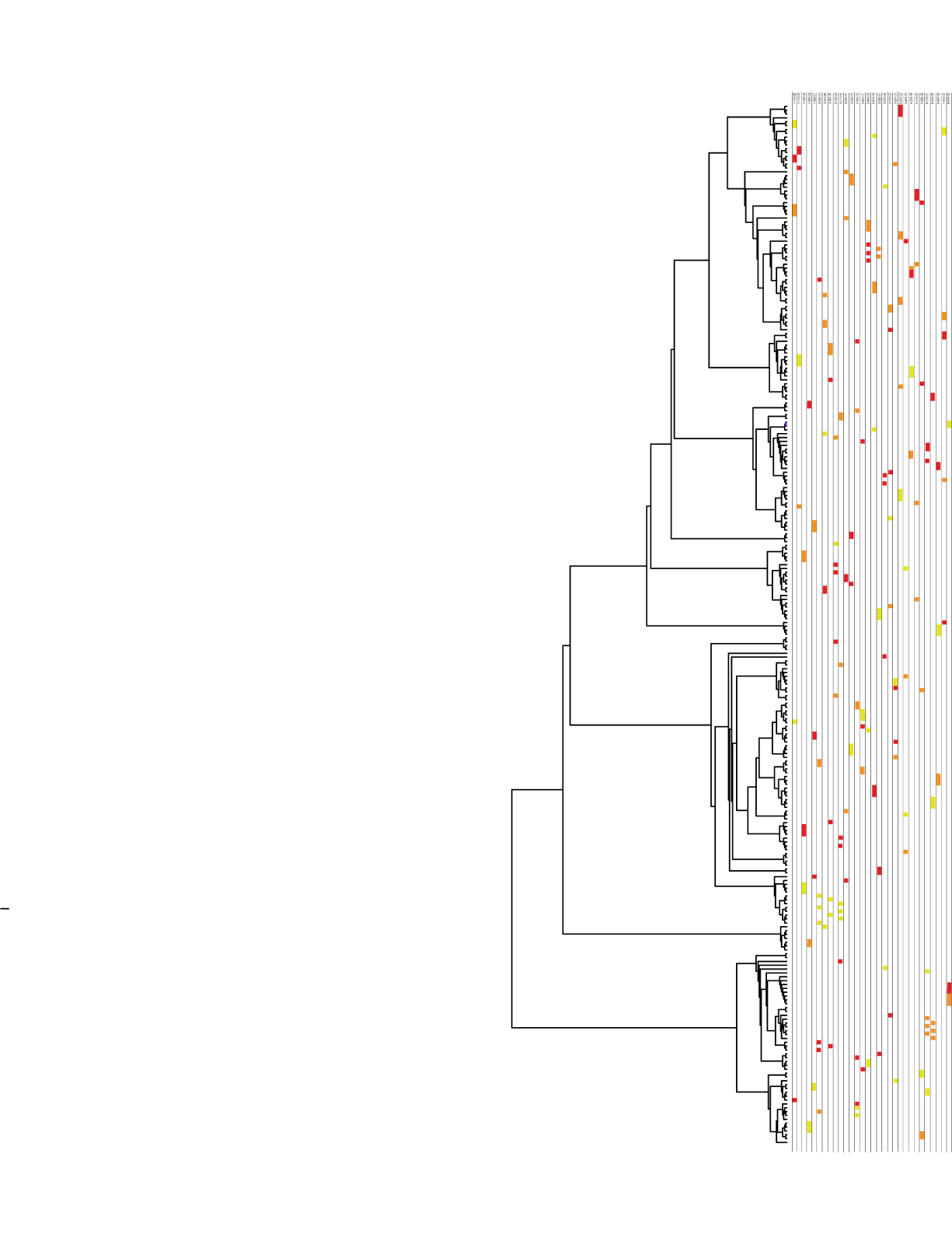

Supplement: FIG S1 [file sph006182690sf1.tif]

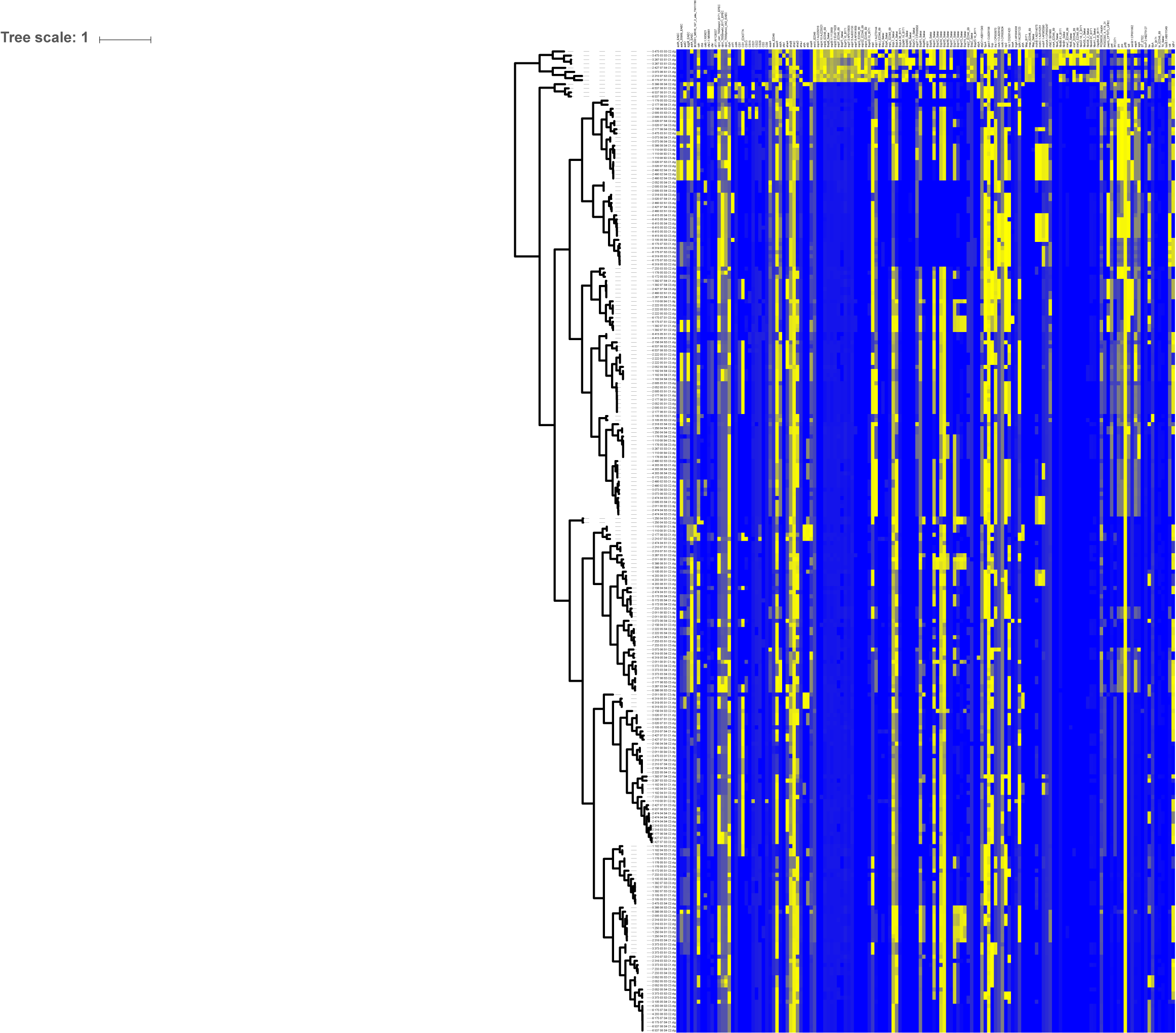

Supplement: FIG S3 [file sph006182690sf3.tif]
