# Supplementary material for: Temporal Variability of Escherichia coli Diversity in the Gastrointestinal Tracts of Tanzanian Children with and without Exposure to Antibiotics
Source: mSphere. 2018 Nov 7;3(6):e00558-18. doi: 10.1128/mSphere.00558-18 (PMC6222053; doi:10.1128/mSphere.00558-18)
Supplement: TABLE S3 [file sph006182690st3.pdf]

Table S3: Table of pairwise gene content comparisons for each individual showing the relatedness of genomes within and across clades.

Subject	Total no. of clades	No. of clades with multiple isolates	No. of isolates in shared clades	*Genomes in shared clades*		*Genomes in different clades*	
				**Average no. of variable genes**	**Standard deviation**	**Average no. variable genes**	**Standard deviation**
1_110_08	5	3	7	64.6	18.6	2597.4	381.1
1_176_05	4	2	6	142.7	69.5	2549	627.8
1_182_04	3	3	9	317.1	435.9	2664.6	432.9
1_250_04	3	3	7	78.4	33.5	2708.1	503.8
1_392_07	4	3	7	95.8	73.3	2942.4	298.3
2_005_03	5	3	7	68.2	26.8	3170.8	565.8
2_011_08	6	2	4	36.5	14.8	2639.3	436.1
2_052_05	5	2	5	99.5	82.9	2781.5	653.1
2_156_04	7	0	0	N/A	N/A	2428.1	433.7
2_177_06	6	2	5	99.3	55.6	2450.3	242.3
2_210_07	6	2	4	66	58	2288.3	409.2
2_222_05	4	3	8	261.9	335.9	2661.6	186.5
2_316_03	6	1	2	42	N/A	2816.6	611.6
2_427_07	5	2	5	628.8	601.5	2501.4	823.4
2_460_02	4	3	8	131.7	106.9	2896	725.4
2_474_04	4	2	6	107.7	84	2433.8	328.5
3_020_07	4	3	7	79	23.6	2653.1	483.6
3_073_06	5	2	4	150.5	92.6	2792.4	455.8
3_105_05	7	2	4	86	65.1	2545.6	642.1
3_267_03	6	1	2	61	N/A	2566.8	391.8
3_373_03	4	3	8	76	32.8	2387.1	252.2
3_475_03	6	0	0	N/A	N/A	2676.2	302.2
4_203_08	3	3	8	88.7	69.6	2008.9	255.9
5_172_05	4	1	3	156.7	35.9	2010.2	280.9
5_366_08	5	2	4	197	69.3	3010.4	210.3
6_175_07	4	3	8	227.6	130.8	3045.9	236.2
6_319_05	3	3	8	285.3	172.5	2968.1	589.4
6_537_08	3	3	8	158.9	60.1	2444.4	214.5
7_233_03	5	3	6	219	177.8	1833.7	377
8_415_05	2	2	8	114.3	65.6	3399.6	57.8
			Mean	147.9	120.1	2629.1	339.4
