# Supplementary material for: Temporal Variability of Escherichia coli Diversity in the Gastrointestinal Tracts of Tanzanian Children with and without Exposure to Antibiotics
Source: mSphere. 2018 Nov 7;3(6):e00558-18. doi: 10.1128/mSphere.00558-18 (PMC6222053; doi:10.1128/mSphere.00558-18)
Supplement: TABLE S4 [file sph006182690st4.pdf]

Table S4: Details of isolate diversity within subject and within time point across several diversity measurements.

	Phylogeny			Phylogroup				MLST				Serotype				Resistance							Virulence					
Sample ID (Subject ID_ Sample No._ Colony No.)	No. clades w/in time point	No. clades across time point	No. clades shared across time points	No. phylogroups w/in time point	Like phylogeny?	No. phylogroups within subject	Like phylogeny?	No. phylogroups w/in time point	Like phylogeny?	No. phylogroups within subject	Like phylogeny?	No. per timepoint	Like phylogeny?	No. per subject	Like phylogeny?	No. clades	Like phylogeny	No. clades across time points	Like phylogeny	Superclade	No. superclades	No. superclades across time point	No. clades	Like phylogeny?	Like resistance?	No. clades across time points	Like phylogeny?	Like resistance?
1_110_08_S1_C1																				1								
1_110_08_S1_C2	2			2	y			2	y			2	y			3	n			1	2		2	y	n			
1_110_08_S1_C3																				2								
1_110_08_S3_C1																				1								
1_110_08_S3_C2	1	5	0	1	y	3	n	1	y	3	n	1	y	5	y	1	y	5	n	1	1	2	1	y	y	5	n	y
1_110_08_S3_C3																				1								
1_110_08_S4_C1																				2								
1_110_08_S4_C2	2			2	y			2	y			2	y			2	y			2	1		2	y	y			
1_110_08_S4_C3																				2								
1_176_05_S1_C1																				2								
1_176_05_S1_C2	1			1	y			1	y			1	y			1	y			2	1		1	y	y			
1_176_05_S1_C3																				2								
1_176_05_S3_C1	2	4	0	2	y	2	n	2	y	3	n	2	y	4	y	2	y	4	y	2	2	2	2	y	y	4	y	y
1_176_05_S3_C2																				1								
1_176_05_S4_C1																				2								
1_176_05_S4_C2	1			1	y			1	y			1	y			1	y			2	1		1	y	y			
1_176_05_S4_C3																				2								
1_182_04_S1_C1																				2								
1_182_04_S1_C2	1			1	y			1	y			1	y			2	n			1	2		1	y	n			
1_182_04_S1_C3																				2								
1_182_04_S3_C1																				2								
1_182_04_S3_C2	1	3	0	1	y	2	n	1	y	3	y	1	y	3	y	2	n	5	n	1	2	2	1	y	n	3	y	n
1_182_04_S3_C3																				2								
1_182_04_S4_C1																				1								
1_182_04_S4_C2	1			1	y			1	y			1	y			3	n			1	1		1	y	n			
1_182_04_S4_C3																				1								
1_250_04_S1_C1																				2								
1_250_04_S1_C2	1			1	y			1	y			1	y			1	y			2	1		1	y	y			
1_250_04_S1_C3																				2								
1_250_04_S3_C1	1	3	0	1	y	3	y	1	y	3	y	1	y	3	y	1	y	2	n	2	1	1	1	y	y	3	y	n
1_250_04_S3_C2																				2								
1_250_04_S4_C1	1			1	y			1	y			1	y			1	y			2	1		1	y	y			
1_250_04_S4_C2																				2								
1_392_07_S1_C1	1			1	y			1	y			1	y			2	n			2	1		1	y	n			
1_392_07_S1_C2																				2								
1_392_07_S3_C1																				1								
1_392_07_S3_C2	1	4	0	1	y	3	n	1	y	4	y	1	y	4	y	1	y	5	n	1	1	2	1	y	y	4	y	n
1_392_07_S3_C3																				1								
1_392_07_S4_C1																				2								
1_392_07_S4_C2	2			1	n			2	y			2	y			2	y			2	1		2	y	y			
1_392_07_S4_C3																				2								
2_005_03_S1_C1																				2								
2_005_03_S1_C2	1			2	n			1	y			1	y			2	y			2	1		2	y	y			
2_005_03_S1_C3																				2								
2_005_03_S3_C1																				1								
2_005_03_S3_C2	2	5	0	2	y	5	y	2	y	5	y	3	n	4	n	2	y	7	n	2	2	2	3	n	n	6	y	n
2_005_03_S3_C3																				1								
2_005_03_S4_C1																				2								
2_005_03_S4_C2	2			2	y			2	y			2	y			3	n			1	2		2	y	n			
2_005_03_S4_C3																				1								
2_011_08_S1_C1																				2								
2_011_08_S1_C2	3			3	y			3	y			3	y			3	y			2	2		3	y	y			
2_011_08_S1_C3																				1								
2_011_08_S3_C1																				2								
2_011_08_S3_C2	2	6	0	1	n	3	n	2	y	6	n	2	y	7	n	3	n	5	n	2	1	2	2	y	n	6	y	n

	Phylogeny			Phylogroup				MLST				Serotype				Resistance							Virulence					
Sample ID (Subject ID_ Sample No._ Colony No.)	No. clades w/in time point	No. clades across time point	No. clades shared across time points	No. phylogroups w/in time point	Like phylogeny?	No. phylogroups within subject	Like phylogeny?	No. phylogroups w/in time point	Like phylogeny?	No. phylogroups within subject	Like phylogeny?	No. per timepoint	Like phylogeny?	No. per subject	Like phylogeny?	No. clades	Like phylogeny	No. clades across time points	Like phylogeny	Superclade	No. superclades	No. superclades across time point	No. clades	Like phylogeny?	Like resistance?	No. clades across time points	Like phylogeny?	Like resistance?
2_011_08_S3_C3																				2								
2_011_08_S4_C1	1			1	n			1	y			2	n			1	y			1	1		1	y	y			
2_011_08_S4_C3																				1								
2_052_05_S1_C1	1			1	y			2	n			2	n			2	n			2	1		1	y	n			
2_052_05_S1_C3																				2								
2_052_05_S3_C1																				1								
2_052_05_S3_C2	1	5	0	1	y	3	n	1	y	5	y	1	y	5	y	1	y	4	y	1	1	2	1	y	y	5	n	n
2_052_05_S3_C3																				1								
2_052_05_S4_C1																				1								
2_052_05_S4_C2	3			3	y			3	y			3	y			3	y			2	2		3	y	y			
2_052_05_S4_C3																				2								
2_156_04_S1_C3	1			1	y			1	y			1	y			1	y			1	1		1	y	y			
2_156_04_S3_C1																				2								
2_156_04_S3_C2	3			2	n			3	y			3	y			3	y			1	2		2	n	n			
2_156_04_S3_C3		7	0			2	n			6	n			7	y			5	y	2		2				6	n	n
2_156_04_S4_C1																				1								
2_156_04_S4_C2	3			2	n			2	n			3	y			3	n			1	1		3	n	y			
2_156_04_S4_C3																				1								
2_177_06_S1_C1																				2								
2_177_06_S1_C2	1			1	y			1	y			1	y			1	y			2	1		2	n	y			
2_177_06_S1_C3																				2								
2_177_06_S3_C1																				2								
2_177_06_S3_C2	2	6	0	2	y	3	n	2	y	6	y	2	y	6	y	2	y	5	n	2	1	2	2	y	y	7	n	n
2_177_06_S3_C3																				2								
2_177_06_S4_C1																				2								
2_177_06_S4_C2	3			2	n			3	y			3	y			3	y			1	2		3	y	y			
2_177_06_S4_C3																				2								
2_210_07_S1_C2	1			1	y			1	y			1	y			2	n			1	1		1	y	n			
2_210_07_S1_C3																				1								
2_210_07_S3_C1																				2								
2_210_07_S3_C2	3	6	0	2	n	2	n	3	y	6	y	3	y	6	y	3	y	6	y	2	2	2	3	y	y	5	n	n
2_210_07_S3_C3																				1								
2_210_07_S4_C1																				2								
2_210_07_S4_C2	2			2	y			2	y			2	y			2	y			1	2		2	y	y			
2_210_07_S4_C3																				1								
2_222_05_S1_C1																				2								
2_222_05_S1_C2	1			1	y			1	y			1	y			1	y			2	1		1	y	y			
2_222_05_S1_C3																				2								
2_222_05_S3_C1																				2								
2_222_05_S3_C2	1	4	0	1	y	2	n	1	y	4	y	1	y	4	y	1	y	5	n	2	1	2	1	y	y	4	y	n
2_222_05_S3_C3																				2								
2_222_05_S4_C1																				1								
2_222_05_S4_C2	2			1	n			2	y			2	y			3	n			1	2		2	y	n			
2_222_05_S4_C3																				2								
2_316_03_S1_C1	1			1	y			1	y			1	y			1	y			2	1		1	y	y			
2_316_03_S1_C2																				2								
2_316_03_S3_C1																				2								
2_316_03_S3_C2	2	6	0	2	y	3	n	2	y	6	y	2	y	5	n	3	n	7	n	1	2	2	2	y	n	5	n	n
2_316_03_S3_C3																				1								
2_316_03_S4_C1																				2								
2_316_03_S4_C2	3			2	n			3	y			3	y			3	y			2	1		3	y	y			
2_316_03_S4_C3																				2								
2_427_07_S1_C1																				1								
2_427_07_S1_C2	1			1	y			1	y			3	n			1	y			1	1		2	n	n			
2_427_07_S1_C3																				1								
2_427_07_S3_C1	1	5	0	1	y	3	n	1	y	5	n	1	y	7	n	1	y	4	y	1	1	2	1	y	y	6	n	n

	Phylogeny			Phylogroup				MLST				Serotype				Resistance							Virulence					
Sample ID (Subject ID_ Sample No._ Colony No.)	No. clades w/in time point	No. clades across time point	No. clades shared across time points	No. phylogroups w/in time point	Like phylogeny?	No. phylogroups within subject	Like phylogeny?	No. phylogroups w/in time point	Like phylogeny?	No. phylogroups within subject	Like phylogeny?	No. per timepoint	Like phylogeny?	No. per subject	Like phylogeny?	No. clades	Like phylogeny	No. clades across time points	Like phylogeny	Superclade	No. superclades	No. superclades across time point	No. clades	Like phylogeny?	Like resistance?	No. clades across time points	Like phylogeny?	Like resistance?
2_427_07_S3_C3	1	[illegible]	[illegible]	1	y	[illegible]	n	1	y	[illegible]	n	1	y	7	n	1	y	4	y	1	1	2	1	y	y	[illegible]	n	n
2_427_07_S4_C1																				2								
2_427_07_S4_C2	3			3	y			3	y			3	y			3	y			2	2		3	y	y			
2_427_07_S4_C3																				1								
2_460_02_S1_C1																				1								
2_460_02_S1_C2	2			2	y			2	y			3	n			2	y			2	2		2	y	y			
2_460_02_S1_C3																				2								
2_460_02_S3_C1																				1								
2_460_02_S3_C2	1	4	0	1	y	3	n	1	y	4	y	1	y	5	n	1	y	4	y	1	1	2	1	y	y	4	y	y
2_460_02_S3_C3																				1								
2_460_02_S4_C1																				2								
2_460_02_S4_C2	1			1	y			1	y			1	y			1	y			2	1		1	y	y			
2_460_02_S4_C3																				2								
2_474_04_S1_C1	2			1	n			2	y			2	y			1	n			1	1		1	n	y			
2_474_04_S1_C2																				1								
2_474_04_S3_C1																				2								
2_474_04_S3_C2	1	4	0	1	y	2	n	1	y	4	y	1	y	4	y	1	n	3	n	2	1	2	1	n	y	3	n	y
2_474_04_S3_C3																				2								
2_474_04_S4_C1																				1								
2_474_04_S4_C2	1			1	y			1	y			1	y			1	y			1	1		1	y	y			
2_474_04_S4_C3																				1								
3_020_07_S1_C1																				1								
3_020_07_S1_C2	1			1	y			1	y			1	y			1	y			1	1		1	y	y			
3_020_07_S1_C3.																				1								
3_020_07_S3_C1	1	4	0	1	y	3	n	1	y	4	y	1	y	4	y	1	y	4	y	2	1	2	1	y	y	4	y	y
3_020_07_S3_C2																				2								
3_020_07_S4_C1																				2								
3_020_07_S4_C2	2			2	y			2	y			2	y			2	y			2	1		2	y	y			
3_020_07_S4_C3																				2								
3_073_06_S1_C1	2			2	y			2	y			2	y			2	y			2	2		2	y	y			
3_073_06_S1_C2																				1								
3_073_06_S3_C1	1			1	y			1	y			1	y			1	y			2	1		1	y	y			
3_073_06_S3_C2		5	0			3	n			5	n			5	n			5	y	2		2				4	n	n
3_073_06_S4_C1																				1								
3_073_06_S4_C2	2			2	y			2	y			2	y			2	y			1	1		2	y	y			
3_073_06_S4_C3																				1								
3_105_05_S1_C1																				1								
3_105_05_S1_C2	2			1	n			2	y			2	y			2	y			2	2		2	y	y			
3_105_05_S1_C3																				1								
3_105_05_S3_C1																				2								
3_105_05_S3_C2	2	7	0	1	n	2	n	2	y	7	y	2	y	7	y	2	y	6	y	2	1	2	2	y	y	7	y	n
3_105_05_S3_C3																				2								
3_105_05_S4_C1																				1								
3_105_05_S4_C2	3			2	n			3	y			3	y			3	y			2	2		3	y	y			
3_105_05_S4_C3																				2								
3_267_03_S1_C1																				1								
3_267_03_S1_C2	2			2	y			2	y			2	y			2	y			1	1		2	y	y			
3_267_03_S1_C3																				1								
3_267_03_S3_C1	2	6	0	2	y	3	n	2	y	6	y	2	y	6	y	2	y	6	y	2	2	2	2	y	y	6	y	y
3_267_03_S3_C2																				1								
3_267_03_S4_C1	2			1	n			2	y			2	y			2	y			2	2		2	y	y			
3_267_03_S4_C2																				1								
3_373_03_S1_C1																				1								
3_373_03_S1_C2	1			1	y			1	y			1	y			1	y			1	1		1	y	y			
3_373_03_S1_C3																				1								
3_373_03_S3_C1																				1								

	Phylogeny			Phylogroup				MLST				Serotype				Resistance							Virulence					
Sample ID (Subject ID_ Sample No._ Colony No.)	No. clades w/in time point	No. clades across time point	No. clades shared across time points	No. phylogroups w/in time point	Like phylogeny?	No. phylogroups within subject	Like phylogeny?	No. phylogroups w/in time point	Like phylogeny?	No. phylogroups within subject	Like phylogeny?	No. per timepoint	Like phylogeny?	No. per subject	Like phylogeny?	No. clades	Like phylogeny	No. clades across time points	Like phylogeny	Superclade	No. superclades	No. superclades across time point	No. clades	Like phylogeny?	Like resistance?	No. clades across time points	Like phylogeny?	Like resistance?
3_373_03_S3_C2	2	4	0	1	n	1	n	2	y	3	n	2	y	4	y	2	y	4	y	2	2	2	2	y	y	3	n	n
3_373_03_S3_C3																				2								
3_373_03_S4_C1																				2								
3_373_03_S4_C2	1			1	y			1	y			1	y			1	y			2	1		1	y	y			
3_373_03_S4_C3																				2								
3_475_03_S1_C1	2			2	y			2	y			2	y			2	y			1	2		2	y	y			
3_475_03_S1_C2																				2								
3_475_03_S3_C1	2	6	0	1	n	2	n	2	y	6	y	2	y	6	y	2	y	5	n	2	2	2	2	y	y	6	y	n
3_475_03_S3_C2																				1								
3_475_03_S4_C1	2			1	n			2	y			2	y			2	y			1	2		2	y	y			
3_475_03_S4_C2																				2								
4_203_08_S1_C1																				2								
4_203_08_S1_C2	1			1	y			1	y			1	y			2	n			2	1		1	y	n			
4_203_08_S1_C3																				2								
4_203_08_S3_C1																				1								
4_203_08_S3_C2	2	3	1	1	n	1	n	1	n	2	n	2	y	3	y	2	y	5	n	1	1	2	2	y	y	3	n	n
4_203_08_S3_C3																				1								
4_203_08_S4_C2	1			1	y			1	y			1	y			2	n			1	2		1	y	n			
4_203_08_S4_C3																				2								
5_172_05_S1_C3	1			1	y			1	y			1	y			1	y			2	1		1	y	y			
5_172_05_S3_C1	2			1	n			2	y			2	y			2	n			1	2		2	y	y			
5_172_05_S3_C3		4	0			1	n			4	y			4	y			3	n	2		2				4	y	n
5_172_05_S4_C1																				1								
5_172_05_S4_C2	1			1	y			1	y			1	y			1	y			1	1		1	y	y			
5_172_05_S4_C3																				1								
5_366_08_S1_C1	1			1	y			1	y			1	y			1	y			2	1		1	y	y			
5_366_08_S1_C3																				2								
5_366_08_S3_C1																				2								
5_366_08_S3_C2	2	5	0	2	y	3	n	2	y	6	n	2	y	5	y	2	y	5	y	2	1	2	2	y	y	4	n	n
5_366_08_S3_C3																				2								
5_366_08_S4_C1	2			1	n			2	y			2	y			2	y			2	2		2	y	y			
5_366_08_S4_C2																				1								
6_175_07_S1_C1																				1								
6_175_07_S1_C2	2			2	y			2	y			2	y			2	y			2	2		2	y	y			
6_175_07_S1_C3																				2								
6_175_07_S3_C1																				1								
6_175_07_S3_C2	1	4	0	1	n	3	n	1	y	4	y	1	y	4	y	2	n	5	y	2	2	2	2	n	y	5	y	y
6_175_07_S3_C3																				2								
6_175_07_S4_C1																				1								
6_175_07_S4_C2	1			1	n			1	y			1	y			2	n			1	1		1	y	n			
6_175_07_S4_C3																				1								
6_319_05_S1_C1																				2								
6_319_05_S1_C2	1			1	y			2	n			1	y			1	y			2	1		1	y	y			
6_319_05_S1_C3																				2								
6_319_05_S3_C1																				2								
6_319_05_S3_C2	1	3	0	1	y	3	y	1	y	4	n	1	y	3	y	2	n	5	n	1	2	2	1	y	n	3	y	n
6_319_05_S3_C3																				2								
6_319_05_S4_C2	1			1	y			1	y			1	y			2	n			1	2		1	y	n			
6_319_05_S4_C3																				2								
6_537_08_S1_C1																				2								
6_537_08_S1_C2	1			1	y			1	y			1	y			2	n			2	1		1	y	n			
6_537_08_S1_C3																				2								
6_537_08_S3_C1																				1								
6_537_08_S3_C2	1	3	0	1	y	2	n	1	y	3	y	1	y	3	y	2	n	5	n	1	1	2	1	y	n	3	y	n
6_537_08_S3_C3																				1								
6_537_08_S4_C1	1			1	y			1	y			1	y			1	y			1	1		1	y	y			

	Phylogeny			Phylogroup				MLST				Serotype				Resistance							Virulence					
Sample ID (Subject ID_ Sample No._ Colony No.)	No. clades w/in time point	No. clades across time point	No. clades shared across time points	No. phylogroups w/in time point	Like phylogeny?	No. phylogroups within subject	Like phylogeny?	No. phylogroups w/in time point	Like phylogeny?	No. phylogroups within subject	Like phylogeny?	No. per timepoint	Like phylogeny?	No. per subject	Like phylogeny?	No. clades	Like phylogeny	No. clades across time points	Like phylogeny	Superclade	No. superclades	No. superclades across time point	No. clades	Like phylogeny?	Like resistance?	No. clades across time points	Like phylogeny?	Like resistance?
6_537_08_S4_C2	1			1	y			1	y			1	y			1	y			1	1		1	y	y			
7_233_03_S1_C2	1	5	0	1	y	1	n	1	y	4	n	1	y	6	n	1	y	5	y	1	1	1	1	y	y	5	y	y
7_233_03_S1_C3																				1								
7_233_03_S3_C1	2			1	n			2	y			3	n			2	y			1	1		2	y	y			
7_233_03_S3_C2																				1								
7_233_03_S3_C3																				1								
7_233_03_S4_C1	2			1	n			2	y			2	y			2	y			1	1		2	y	y			
7_233_03_S4_C2																				1								
7_233_03_S4_C3																				1								
8_415_05_S1_C1	1	2	1	1	y	2	y	1	y	3	n	1	y	2	y	2	n	3	n	2	2	2	1	y	n	2	y	n
8_415_05_S1_C2																				1								
8_415_05_S3_C1	1			1	y			1	y			1	y			1	y			2	1		1	y	y			
8_415_05_S3_C2																				2								
8_415_05_S3_C3																				2								
8_415_05_S4_C1	1			1	y			2	n			1	y			1	y			2	1		1	y	n			
8_415_05_S4_C2																				2								
8_415_05_S4_C3																				2								
Total in common with phylogeny					65		4		85		18		84		23													
