# Supplementary material for: Temporal Variability of Escherichia coli Diversity in the Gastrointestinal Tracts of Tanzanian Children with and without Exposure to Antibiotics
Source: mSphere. 2018 Nov 7;3(6):e00558-18. doi: 10.1128/mSphere.00558-18 (PMC6222053; doi:10.1128/mSphere.00558-18)
Supplement: TABLE S5 [file sph006182690st5.pdf]

Table S5. Examples of isolates with identical serotypes, but differing sequence type and/or phylotype

	Isolate	MLST	Phylotype	Serotype
Shared serotype, different MLST and phylotype	6_175_07_S1_C2	38	D	O153var1:H2
	6_175_07_S1_C3	38	D	O153var1:H2
	3_267_03_S3_C2	101	B1	O153var1:H2
	2_005_03_S4_C2	452	B2	O81:H27
	2_005_03_S4_C3	452	B2	O81:H27
	2_005_03_S3_C1	2178	B1	O81:H27
	2_005_03_S3_C3	2178	B1	O81:H27
Shared serotype and phylotype, different MLST	1_110_08_S3_C1	10	A	O176:H33
	1_110_08_S3_C2	10	A	O176:H33
	1_110_08_S3_C3	10	A	O176:H33
	5_366_08_S4_C1	5793	A	O176:H33
	3_105_05_S1_C2	206	A	O60:H5
	5_172_05_S3_C3	206	A	O60:H5
	3_373_03_S1_C1	5293	A	O60:H5
	3_373_03_S1_C2	5293	A	O60:H5
	3_373_03_S1_C3	5293	A	O60:H5
Shared serotype and MLST, different phylotype	2_005_03_S1_C1	5257	B1	O8:H12
	2_005_03_S1_C2	5257	B1	O8:H12
	2_005_03_S1_C3	5257	E	O8:H12
	2_052_05_S1_C1	5257	B1	O8:H12
	2_177_06_S1_C1	5257	B1	O8:H12
	2_177_06_S1_C2	5257	B1	O8:H12
	2_177_06_S1_C3	5257	B1	O8:H12
