# Supplementary material for: Temporal Variability of Escherichia coli Diversity in the Gastrointestinal Tracts of Tanzanian Children with and without Exposure to Antibiotics
Source: mSphere. 2018 Nov 7;3(6):e00558-18. doi: 10.1128/mSphere.00558-18 (PMC6222053; doi:10.1128/mSphere.00558-18)
Supplement: TABLE S7 [file sph006182690st7.pdf]

Table S7: Presence of Macrolide Resistance Genes

	mphB	*mphA*	*mphC*	*ermG*	total in timepoint with resistance genes	%	pre vs post	%
1_110_08_S1_C1	0	0	0	0.13	0	0	0	0
1_110_08_S1_C2	0	0	0	0.13				
1_110_08_S1_C3	0	0	0	0.13				
1_110_08_S3_C1	0	0	0	0.13	0	0	3	50
1_110_08_S3_C2	0	0	0	0.13				
1_110_08_S3_C3	0	0	0	0.13				
1_110_08_S4_C1	0.33	1	0.31	0.13	3	100		
1_110_08_S4_C2	0.33	1	0.31	0.13				
1_110_08_S4_C3	0.33	1	0.31	0.13				
1_176_05_S1_C1	0	0	0	0.13	0	0	0	0
1_176_05_S1_C2	0	0	0	0.13				
1_176_05_S1_C3	0	0	0	0.13				
1_176_05_S3_C1	0.33	1	0.31	0.13	1	50	4	80
1_176_05_S3_C2	0	0	0	0.13				
1_176_05_S4_C1	0.33	1	0.31	0.13	3	100		
1_176_05_S4_C2	0.33	1	0.31	0.13				
1_176_05_S4_C3	0.33	1	0.31	0.13				
1_182_04_S1_C1	0	0	0	0.13	0	0	0	0
1_182_04_S1_C2	0	0	0	0.13				
1_182_04_S1_C3	0	0.06	0	0.13				
1_182_04_S3_C1	0.33	1	0.31	0.13	2	66	2	33
1_182_04_S3_C2	0	0	0	0.07				
1_182_04_S3_C3	0.33	1	0.31	0.13				
1_182_04_S4_C1	0	0	0	0.13	0	0		
1_182_04_S4_C2	0	0	0	0.13				
1_182_04_S4_C3	0	0	0	0.13				
1_250_04_S1_C1	0.33	1	0.31	0.13	3	100	3	100
1_250_04_S1_C2	0.33	0.98	0.31	0.13				
1_250_04_S1_C3	0.33	1	0.31	0.13				
1_250_04_S3_C1	0.33	1	0.31	0.13	2	100	4	100
1_250_04_S3_C2	0.33	1	0.31	0.12				
1_250_04_S4_C1	0.33	1	0.31	0.13	2	100		
1_250_04_S4_C2	0.33	1	0.31	0.13				
1_392_07_S1_C1	0	0	0	0.13	0	0	0	0
1_392_07_S1_C2	0	0	0	0.13				
1_392_07_S3_C1	0	0	0	0.13	0	0	2	33
1_392_07_S3_C2	0	0	0	0.13				
1_392_07_S3_C3	0	0	0	0.13				
1_392_07_S4_C1	0.33	1	0.31	0.13	2	66		
1_392_07_S4_C2	0	0	0	0.13				
1_392_07_S4_C3	0.33	1	0.31	0.13				
2_005_03_S1_C1	0	0.06	0	0.13	0	0	0	0
2_005_03_S1_C2	0	0.06	0	0.13				
2_005_03_S1_C3	0	0.06	0	0.13				
2_005_03_S3_C1	0.33	1	0.31	0.13	2	66	2	33
2_005_03_S3_C2	0	0	0	0.13				
2_005_03_S3_C3	0.33	1	0.31	0.13				
2_005_03_S4_C1	0	0	0	0.13	0	0		
2_005_03_S4_C2	0	0	0	0.13				
2_005_03_S4_C3	0	0	0	0.13				
2_011_08_S1_C1	0	0	0	0.13				

	mphB	*mphA*	*mphC*	*ermG*	total in timepoint with resistance genes	%	pre vs post	%
2_011_08_S1_C2	0	0	0	0.13	0	0	0	0
2_011_08_S1_C3	0	0	0	0.13				
2_011_08_S3_C1	0.33	1	0.31	0.13	2	66	2	40
2_011_08_S3_C2	0	0	0	0.13				
2_011_08_S3_C3	0.33	1	0.31	0.13				
2_011_08_S4_C1	0	0	0	0.13	0	0		
2_011_08_S4_C3	0	0	0	0.13				
2_052_05_S1_C1	0	0.06	0	0.13	0	0	0	0
2_052_05_S1_C3	0	0.06	0	0.13				
2_052_05_S3_C1	0	0	0	0.13	0	0	0	0
2_052_05_S3_C2	0	0	0	0.13				
2_052_05_S3_C3	0	0	0	0.13				
2_052_05_S4_C1	0	0	0	0.13	0	0		
2_052_05_S4_C2	0	0	0	0.13				
2_052_05_S4_C3	0	0	0	0.13				
2_156_04_S1_C3	0	0	0	0.13	0	0	0	0
2_156_04_S3_C1	0	0	0	0.13	0	0	0	0
2_156_04_S3_C2	0	0	0	0.13				
2_156_04_S3_C3	0	0	0	0.13				
2_156_04_S4_C1	0	0	0	0.13	0	0		
2_156_04_S4_C2	0	0	0	0.13				
2_156_04_S4_C3	0	0	0	0.13				
2_177_06_S1_C1	0	0.06	0	0.13	0	0	0	0
2_177_06_S1_C2	0	0.06	0	0.13				
2_177_06_S1_C3	0	0.06	0	0.13				
2_177_06_S3_C1	0	0	0	0.13	0	0	0	0
2_177_06_S3_C2	0	0	0	0.13				
2_177_06_S3_C3	0	0	0	0.13				
2_177_06_S4_C1	0	0	0	0.13	0	0		
2_177_06_S4_C2	0	0	0	0.13				
2_177_06_S4_C3	0	0	0	0.13				
2_210_07_S1_C2	0	0	0	0.13	0	0	0	0
2_210_07_S1_C3	0	0	0	0.13				
2_210_07_S3_C1	0.33	0.99	0.31	0.13	1	33	1	16.5
2_210_07_S3_C2	0	0	0	0.13				
2_210_07_S3_C3	0	0	0	0.13				
2_210_07_S4_C1	0	0	0	0.13	0	0		
2_210_07_S4_C2	0	0	0	0.13				
2_210_07_S4_C3	0	0	0	0.13				
2_222_05_S1_C1	0	0	0	0.13	0	0	0	0
2_222_05_S1_C2	0	0	0	0.13				
2_222_05_S1_C3	0	0	0	0.13				
2_222_05_S3_C1	0	0	0	0.13	0	0	0	0
2_222_05_S3_C2	0	0	0	0.13				
2_222_05_S3_C3	0	0	0	0.13				
2_222_05_S4_C1	0	0	0	0.13	0	0		
2_222_05_S4_C2	0	0	0	0.13				
2_222_05_S4_C3	0	0	0	0.13				
2_316_03_S1_C1	0	0	0	0.13	0	0	0	0
2_316_03_S1_C2	0	0	0	0.13				
2_316_03_S3_C1	0	0	0	0.13	0	0		
2_316_03_S3_C2	0	0	0	0.13				

	mphB	*mphA*	*mphC*	*ermG*	total in timepoint with resistance genes	%	pre vs post	%
2_316_03_S3_C3	0	0	0	0.13			2	33
2_316_03_S4_C1	0.33	1	0.31	0.13				
2_316_03_S4_C2	0.33	1	0.31	0.13	2	66		
2_316_03_S4_C3	0	0	0	0.13				
2_427_07_S1_C1	0	0	0	0.13				
2_427_07_S1_C2	0	0	0	0.13	0	0	0	0
2_427_07_S1_C3	0	0	0	0.13				
2_427_07_S3_C1	0	0	0	0.13	0	0		
2_427_07_S3_C3	0	0	0	0.13				
2_427_07_S4_C1	0.33	1	0.31	0.13			2	40
2_427_07_S4_C2	0.33	1	0.31	0.13	2	66		
2_427_07_S4_C3	0	0	0	0.13				
2_460_02_S1_C1	0	0	0	0.13				
2_460_02_S1_C2	0.33	1	0.31	0.13	2	66	2	66
2_460_02_S1_C3	0.33	1	0.31	0.13				
2_460_02_S3_C1	0	0	0	0.13				
2_460_02_S3_C2	0	0	0	0.13	0	0		
2_460_02_S3_C3	0	0	0	0.13				
2_460_02_S4_C1	0.33	1	0.31	0.13			3	50
2_460_02_S4_C2	0.33	1	0.31	0.13	3	100		
2_460_02_S4_C3	0.33	1	0.31	0.13				
2_474_04_S1_C1	0	0	0	0.13	0	0	0	0
2_474_04_S1_C2	0	0	0	0.13				
2_474_04_S3_C1	0	0	0	0.13				
2_474_04_S3_C2	0	0	0	0.13	0	0		
2_474_04_S3_C3	0	0	0	0.13				
2_474_04_S4_C1	0	0	0	0.13			0	0
2_474_04_S4_C2	0	0	0	0.13	0	0		
2_474_04_S4_C3	0	0	0	0.13				
3_020_07_S1_C1	0	0	0	0.13				
3_020_07_S1_C2	0	0	0	0.13	0	0	0	0
3_020_07_S1_C3	0	0	0	0.13				
3_020_07_S3_C1	0.33	1	0.31	0.13	2	100		
3_020_07_S3_C2	0.33	1	0.31	0.13				
3_020_07_S4_C1	0	0	0	0.13			4	80
3_020_07_S4_C2	0.33	1	0.31	0.13	2	66		
3_020_07_S4_C3	0.33	1	0.31	0.13				
3_073_06_S1_C1	0	0	0	0.13	0	0	0	0
3_073_06_S1_C2	0	0	0	0.13				
3_073_06_S3_C1	0	0	0	0.13	0	0		
3_073_06_S3_C2	0	0	0	0.13				
3_073_06_S4_C1	0	0	0	0.13			0	0
3_073_06_S4_C2	0	0	0	0.13	0	0		
3_073_06_S4_C3	0	0	0	0.13				
3_105_05_S1_C1	0	0	0	0.13				
3_105_05_S1_C2	0.33	1	0.31	0.48	1	33	1	33
3_105_05_S1_C3	0	0	0	0.13				
3_105_05_S3_C1	0	0	0	0.13				
3_105_05_S3_C2	0	0	0	0.13	1	33		
3_105_05_S3_C3	0.33	1	0.31	0.13			2	33
3_105_05_S4_C1	0	0	0	0.13				
3_105_05_S4_C2	0	0	0	0.13	1	33		

	mphB	*mphA*	*mphC*	*ermG*	total in timepoint with resistance genes	%	pre vs post	%
3_105_05_S4_C3	0.33	1	0.31	0.13				
3_267_03_S1_C1	0	0	0	0.13	0	0	0	0
3_267_03_S1_C2	0	0	0	0.13				
3_267_03_S1_C3	0	0	0	0.13				
3_267_03_S3_C1	0.33	1	0.31	0.13	1	50	2	50
3_267_03_S3_C2	0	0	0	0.13				
3_267_03_S4_C1	0.33	1	0.31	0.13	1	50		
3_267_03_S4_C2	0	0	0	0.13				
3_373_03_S1_C1	0	0	0	0.13	0	0	0	0
3_373_03_S1_C2	0	0	0	0.13				
3_373_03_S1_C3	0	0	0	0.13				
3_373_03_S3_C1	0	0	0	0.13	0	0	0	0
3_373_03_S3_C2	0	0	0	0.13				
3_373_03_S3_C3	0	0	0	0.13				
3_373_03_S4_C1	0	0	0	0.13	0	0		
3_373_03_S4_C2	0	0	0	0.13				
3_373_03_S4_C3	0	0	0	0.13				
3_475_03_S1_C1	0	0	0	0.13	0	0	0	0
3_475_03_S1_C2	0	0	0	0.13				
3_475_03_S3_C1	0	0	0	0.13	0	0	0	0
3_475_03_S3_C2	0	0	0	0.13				
3_475_03_S4_C1	0	0	0	0.13	0	0		
3_475_03_S4_C2	0	0	0	0.13				
4_203_08_S1_C1	0	0	0	0.13	0	0	0	0
4_203_08_S1_C2	0	0	0	0.13				
4_203_08_S1_C3	0	0	0	0.13				
4_203_08_S3_C1	0	0	0	0.13	0	0	1	20
4_203_08_S3_C2	0	0	0	0.13				
4_203_08_S3_C3	0	0	0	0.13				
4_203_08_S4_C2	0	0.13	0	0.13	1	50		
4_203_08_S4_C3	0.25	0.72	0.23	0.13				
5_172_05_S1_C3	0.33	1	0.31	0.13	1	100	1	100
5_172_05_S3_C1	0	0	0	0.13	0	0	0	0
5_172_05_S3_C3	0	0	0	0.13				
5_172_05_S4_C1	0	0	0	0.13	0	0		
5_172_05_S4_C2	0	0	0	0.13				
5_172_05_S4_C3	0	0	0	0.13				
5_366_08_S1_C1	0	0	0	0.13	0	0	0	0
5_366_08_S1_C3	0	0	0	0.13				
5_366_08_S3_C1	0.33	1	0.31	0.13	2	66	2	40
5_366_08_S3_C2	0	0	0	0.13				
5_366_08_S3_C3	0.33	1	0.31	0.13				
5_366_08_S4_C1	0	0	0	0.13	0	0		
5_366_08_S4_C2	0	0	0	0.13				
6_175_07_S1_C1	0	0	0	0.13	0	0	0	0
6_175_07_S1_C2	0	0	0	0.13				
6_175_07_S1_C3	0	0	0	0.13				
6_175_07_S3_C1	0	0	0	0.13	0	0	0	0
6_175_07_S3_C2	0	0	0	0.13				
6_175_07_S3_C3	0	0	0	0.13				
6_175_07_S4_C1	0	0	0	0.13	0	0		
6_175_07_S4_C2	0	0	0	0.13				

	mphB	*mphA*	*mphC*	*ermG*	total in timepoint with resistance genes	%	pre vs post	%
6_175_07_S4_C3	0	0	0	0.13				
6_319_05_S1_C1	0	0	0	0.13	0	0	0	0
6_319_05_S1_C2	0	0	0	0.13				
6_319_05_S1_C3	0	0	0	0.13				
6_319_05_S3_C1	0	0	0	0.13	0	0	0	0
6_319_05_S3_C2	0	0	0	0.13				
6_319_05_S3_C3	0	0	0	0.13				
6_319_05_S4_C2	0	0	0	0.13	0	0		
6_319_05_S4_C3	0	0	0	0.13				
6_537_08_S1_C1	0	0	0	0.13	0	0	0	0
6_537_08_S1_C2	0	0	0	0.13				
6_537_08_S1_C3	0.17	0.55	0.15	0.13				
6_537_08_S3_C1	0	0	0	0.13	0	0	0	0
6_537_08_S3_C2	0	0	0	0.13				
6_537_08_S3_C3	0	0	0	0.13				
6_537_08_S4_C1	0	0	0	0.13	0	0		
6_537_08_S4_C2	0	0	0	0.13				
7_233_03_S1_C2	0	0	0	0.13	0	0	0	0
7_233_03_S1_C3	0	0	0	0.13				
7_233_03_S3_C1	0.33	1	0.31	0.13	2	66	2	40
7_233_03_S3_C2	0	0	0	0.13				
7_233_03_S3_C3	0.32	0.98	0.3	0.13				
7_233_03_S4_C1	0	0	0	0.13	0	0		
7_233_03_S4_C2	0	0	0	0.13				
7_233_03_S4_C3	0	0	0	0.13				
8_415_05_S1_C1	0	0	0	0.13	0	0	0	0
8_415_05_S1_C2	0	0	0	0.13				
8_415_05_S3_C1	0	0	0	0.13	0	0	0	0
8_415_05_S3_C2	0	0	0	0.13				
8_415_05_S3_C3	0	0	0	0.13				
8_415_05_S4_C1	0	0	0	0.13	0	0		
8_415_05_S4_C2	0	0	0	0.13				
8_415_05_S4_C3	0	0	0	0.13				
